# Supplementary material for: Nim1-related kinases regulate septin organization and cytokinesis by modulating Hof1 at the cell division site
Source: eLife. 2026 Sep 9;14:RP106366. doi: 10.7554/eLife.106366 (PMC13557712; doi:10.7554/eLife.106366)
Supplement: Supplementary file 1 [file elife-106366-mdarchecklist1.docx]

**Materials Design Analysis Reporting (MDAR)**

**Checklist for Authors**

The [MDAR framework](https://osf.io/xfpn4/) establishes a minimum set of requirements in transparent reporting mainly applicable to studies in the life sciences.

*eLife* asks authors to **provide detailed information within their article** to facilitate the interpretation and replication of their work. Authors can also upload supporting materials to comply with relevant reporting guidelines for health-related research (see [EQUATOR Network](http://www.equator-network.org/%20)), life science research (see the [BioSharing Information Resource](http://biosharing.org/)), or animal research (see the [ARRIVE Guidelines](http://www.plosbiology.org/article/info:doi/10.1371/journal.pbio.1000412) and the [STRANGE Framework](https://doi.org/10.1038/d41586-020-01751-5); for details, see *eLife*’s [Journal Policies](https://reviewer.elifesciences.org/author-guide/journal-policies)). Where applicable, authors should refer to any relevant reporting standards materials in this form.

For all that apply, please note **where in the article** the information is provided. Please note that we also collect information about data availability and ethics in the submission form.

**Materials:**

| **Newly created materials** | **Lead contact and materials availability** | **N/A** |
| --- | --- | --- |
| The manuscript includes a dedicated "materials availability statement" providing transparent disclosure about availability of newly created materials including details on how materials can be accessed and describing any restrictions on access. | Further information and requests for resources and reagents should be directed to and will be fulfilled by the lead contact, Dr. Saravanan Palani ([spalani@iisc.ac.in](mailto:spalani@iisc.ac.in)).  Plasmids and yeast strains generated in this study are available from the lead contact upon request. |  |
|  |  |  |
| **Antibodies** | **Key resource table, Materials and Methods- In vitro binding assay** | **N/A** |
| For commercial reagents, provide supplier name, catalogue number and [RRID](https://scicrunch.org/resources), if available. | The antibodies used for in vitro binding experiments are listed in the Key resource table with the company name and Lot/catalog. number.  Anti-His (H-3) mouse monoclonal IgG1 (Santa Cruz Biotechnology; Cat.#sc-8036, Lot#C0421)  Anti-GST (B-14) mouse monoclonal IgG1 (Santa Cruz Biotechnology; Cat.#sc-138, Lot#K1020)  Anti-mouse IgG-HRP (Cell Signalling Technology; Cat.#7076S, Lot#36) |  |
|  |  |  |
| **DNA and RNA sequences** | **Supplementary Table S2 and S3; Materials and Methods-Oligonucleotides and Plasmids used in the study** | **N/A** |
| Short novel DNA or RNA including primers, probes: Sequences should be included or deposited in a public repository. | Primers used for endogenous gene tagging, deletion, cloning and mutagenesis are listed in the supplementary table S2.  Plasmids used in the study are listed in supplementary table S3 with appropriate citations. |  |
|  |  |  |
| **Cell materials** | **N/A** | **N/A** |
| Cell lines: Provide species information, strain. Provide accession number in repository OR supplier name, catalog number, clone number, OR RRID. |  |  |
| Primary cultures: Provide species, strain, sex of origin, genetic modification status. |  | N/A |
|  |  |  |
| **Experimental animals** | **N/A** | **N/A** |
| Laboratory animals or Model organisms: Provide species, strain, sex, age, genetic modification status. Provide accession number in repository OR supplier name, catalog number, clone number, OR RRID. |  |  |
| Animal observed in or captured from the field: Provide species, sex, and age where possible. |  | N/A |
|  |  |  |
| **Plants and microbes** | **Key resource table; Supplementary table S1; Materials and methods-strain construction** | **N/A** |
| Plants: provide species and strain, ecotype and cultivar where relevant, unique accession number if available, and source (including location for collected wild specimens). |  | N/A |
| Microbes: provide species and strain, unique accession number if available, and source. | *Saccharomyces cerevisiae* strains used in this study were constructed in ESM356 and YPH499 wild-type genetic backgrounds derived from S288C. Strain numbers, genotypes and appropriate sources are provided in the Supplementary table S1.  Newly generated gene deletion and epitope tagging strains were constructed in our laboratory using PCR-based integration, as described and cited in the materials and methods section. |  |
|  |  |  |
| **Human research participants** | **N/A** | **N/A** |
| If collected and within the bounds of privacy constraints report on age, sex, gender and ethnicity for all study participants. |  | N/A |

**Design:**

| **Study protocol** | **N/A** | **N/A** |
| --- | --- | --- |
| If the study protocol has been pre-registered, provide DOI. For clinical trials, provide the trial registration number OR cite DOI. |  | N/A |
|  |  |  |
| **Laboratory protocol** | **Indicate where provided: section/figure legend** | **N/A** |
| Provide DOI OR other citation details if detailed step-by-step protocols are available. | Established procedure and protocols used in this study are cited in the materials and methods section where applicable, including PCR-based integration, Lithium acetate mediated yeast transformation, image-analysis workflows and yeast-two hybrid assays. | N/A |
|  |  |  |
| **Experimental study design (statistics details) *** | | |
| **For in vivo studies: State whether and how the following have been done** | **Materials and methods-statistical analysis; figure legends** | **N/A** |
| Sample size determination | Sample sizes (n) are reported in the corresponding quantification graphs or figure legends. No formal power analysis was performed. Sample sizes were selected based on the established practices for quantitative live-cell imaging and cell-based analyses.  A minimum of 15-20 cells were analyzed for quantifying protein kinetics in the live-cell imaging experiments, whereas at least 100 cells were analyzed for quantification of cellular morphology and protein misorganization. |  |
| Randomisation | Not applicable (Experiments did not involve allocation of animals or human participants to treatment groups). |  |
| Blinding | Not done. |  |
| Inclusion/exclusion criteria | All measurable samples were included in the analysis. |  |
|  |  |  |
| **Sample definition and in-laboratory replication** | **Materials and methods-statistical analysis; figure legends** | **N/A** |
| State number of times the experiment was replicated in the laboratory. | All experiments were performed independently three times, unless otherwise indicated in the manuscript. |  |
| Define whether data describe technical or biological replicates. | Data represent independent biological replicates (N). The number of cells analyzed (n) for the quantification is indicated in the corresponding figures or figure legends. |  |
|  |  |  |
| **Ethics** | **N/A** | **N/A** |
| Studies involving human participants: State details of authority granting ethics approval (IRB or equivalent committee(s), provide reference number for approval. |  | N/A |
| Studies involving experimental animals: State details of authority granting ethics approval (IRB or equivalent committee(s), provide reference number for approval. |  | N/A |
| Studies involving specimen and field samples: State if relevant permits obtained, provide details of authority approving study; if none were required, explain why. |  | N/A |
|  |  |  |
| **Dual Use Research of Concern (DURC)** | **N/A** | **N/A** |
| If study is subject to dual use research of concern regulations, state the authority granting approval and reference number for the regulatory approval. |  | N/A |

**Analysis:**

| **Attrition** | **Materials and methods-Quantitative analyses, statistical analysis** | **N/A** |
| --- | --- | --- |
| Describe whether exclusion criteria were pre-established. Report if sample or data points were omitted from analysis. If yes, report if this was due to attrition or intentional exclusion and provide justification. | All measurable samples were included in the analysis.  No samples or data points were intentionally excluded. |  |
|  |  |  |
| **Statistics** | **Materials and methods-statistical analysis, figure legends** | **N/A** |
| Describe statistical tests used and justify choice of tests. | Statistical significance for bar graphs and stacked column plots was assessed using one-way ANOVA with Tukey’s multiple-comparison test. The data involving the residence time were analyzed using the Kruskal-Wallis nonparametric test.  Statistical analyses were performed in GraphPad Prism v6.04. A p-value corresponding to <0.05 was considered statistically significant. The number of cells analyzed is reported with the corresponding figures or figure legends. |  |
|  |  |  |
| **Data availability** | **Data availability section** | **N/A** |
| For newly created and reused datasets, the manuscript includes a data availability statement that provides details for access (or notes restrictions on access). | All data generated or analyzed during this study are included in the manuscript and its supporting files.  Source data files containing the numerical data underlying the quantification in figures and figure supplements, as well as the uncropped western blot images underlying the in vitro binding experiments have been provided. |  |
| When newly created datasets are publicly available, provide accession number in repository OR DOI and licensing details where available. |  |  |
| If reused data is publicly available provide accession number in repository OR DOI, OR URL, OR citation. |  | N/A |
|  |  |  |
| **Code availability** | **N/A** | **N/A** |
| For any computer code/software/mathematical algorithms essential for replicating the main findings of the study, whether newly generated or re-used, the manuscript includes a data availability statement that provides details for access or notes restrictions. |  | N/A |
| Where newly generated code is publicly available, provide accession number in repository, OR DOI OR URL and licensing details where available. State any restrictions on code availability or accessibility. |  | N/A |
| If reused code is publicly available provide accession number in repository OR DOI OR URL, OR citation. |  | N/A |

**Reporting:**

The MDAR framework recommends adoption of discipline-specific guidelines, established and endorsed through community initiatives.

| **Adherence to community standards** | **N/A** | **N/A** |
| --- | --- | --- |
| State if relevant guidelines (e.g., ICMJE, MIBBI, ARRIVE, STRANGE) have been followed, and whether a checklist (e.g., CONSORT, PRISMA, ARRIVE) is provided with the manuscript. |  | N/A |

* We provide the following guidance regarding transparent reporting and statistics; we also refer authors to [Ten common statistical mistakes to watch out for when writing or reviewing a manuscript](https://doi.org/10.7554/eLife.48175).

**Sample-size estimation**

- You should state whether an appropriate sample size was computed when the study was being designed
- You should state the statistical method of sample size computation and any required assumptions
- If no explicit power analysis was used, you should describe how you decided what sample (replicate) size (number) to use

**Replicates**

- You should report how often each experiment was performed
- You should include a definition of biological versus technical replication
- The data obtained should be provided and sufficient information should be provided to indicate the number of independent biological and/or technical replicates
- If you encountered any outliers, you should describe how these were handled
- Criteria for exclusion/inclusion of data should be clearly stated
- High-throughput sequence data should be uploaded before submission, with a private link for reviewers provided (these are available from both GEO and ArrayExpress)

**Statistical reporting**

- Statistical analysis methods should be described and justified
- Raw data should be presented in figures whenever informative to do so (typically when N per group is less than 10)
- For each experiment, you should identify the statistical tests used, exact values of N, definitions of center, methods of multiple test correction, and dispersion and precision measures (e.g., mean, median, SD, SEM, confidence intervals; and, for the major substantive results, a measure of effect size (e.g., Pearson's r, Cohen's d)
- Report exact p-values wherever possible alongside the summary statistics and 95% confidence intervals. These should be reported for all key questions and not only when the p-value is less than 0.05.

**Group allocation**

- Indicate how samples were allocated into experimental groups (in the case of clinical studies, please specify allocation to treatment method); if randomization was used, please also state if restricted randomization was applied
- Indicate if masking was used during group allocation, data collection and/or data analysis
