# Supplementary material for: Nim1-related kinases regulate septin organization and cytokinesis by modulating Hof1 at the cell division site
Source: eLife. 2026 Sep 9;14:RP106366. doi: 10.7554/eLife.106366 (PMC13557712; doi:10.7554/eLife.106366)
Supplement: Supplementary file 2 [file elife-106366-supp1.docx]

**Yeast strains used in this study:**

| **Strain number** | **Genotype** | **Source** |
| --- | --- | --- |
| YSP002 | *MATa ura3-52 leu2∆1 trp1∆63 his3∆200 (ESM356)* | ^1^ |
| YSP120 | *ESM356-Elm1-GFP-Hph Cdc3-mCherry-Trp* | This study |
| YSP121 | *ESM356-Gin4-GFP-Hph Cdc3-mCherry-Trp* | This study |
| YSP122 | *ESM356-Kcc4-GFP-Hph Cdc3-mCherry-Trp* | This study |
| YSP123 | *ESM356-Hsl1-GFP-Hph Cdc3-mCherry-Trp* | This study |
| YSP524 | *ESM356-Cdc3-mCherry-Trp, Tub1-GFP-ura* | This study |
| YSP520 | *ESM356-Cdc3-mCherry-Trp, Tub1-GFP-Ura, ∆elm1::His3mX6* | This study |
| YSP521 | *ESM356-Cdc3-mCherry-Trp, Tub1-GFP-Ura, ∆gin4::His3mX6* | This study |
| YSP1104 | *ESM356-Cdc3-mCherry-Trp, Tub1-GFP-Ura, ∆kcc4::His3mX6* | This study |
| YSP518 | *ESM356-∆hsl1-His3mX6, Cdc3-mCherry-Trp, Tub1-GFP-Ura* | This study |
| YSP058 | *ESM356-∆elm1::His3MX6* | This study |
| YSP062 | *ESM356-∆gin4::His3MX6* | This study |
| YSP050 | *ESM356-∆hsl1::His3MX6* | This study |
| YSP115 | *ESM356-∆kcc4::KanMX6* | This study |
| YSP950 | *ESM356-Inn1-mNG-KanMX6, pHis3-mRuby2-Tub1-Ura3* | This study |
| YSP1017 | *ESM356-Inn1-mNG-KanMX6, pHis3-mRuby2-Tub1-Ura3, ∆elm1::3HA-Trp* | This study |
| YSP1004 | *ESM356-Inn1-mNG-KanMX6, pHis3-mRuby2-Tub1-Ura3, ∆gin4::3HA-Trp* | This study |
| YSP1014 | *ESM356-Inn1-mNG-KanMX6, pHis3-mRuby2-Tub1-Ura3, ∆hsl1::3HA-Trp* | This study |
| YSP1037 | *ESM356-Inn1-mNG-Kanmx6, pHis3-mRuby2-Tub1-Ura3, ∆kcc4::3HA-Trp* | This study |
| YSP948 | *ESM356-Hof1-mNG-KanMX6, pHis3-mRuby2-Tub1-Ura3* | This study |
| YSP1024 | *ESM356-Hof1-mNG-KanMX6, pHis3-mRuby2-Tub1-Ura3, ∆elm1::3HA-Trp* | This study |
| YSP1095 | *ESM356-Hof1-mNG-Kanmx6, pHis3-mRuby2-Tub1-Ura3, ∆gin4::3HA-Trp* | This study |
| YSP947 | *ESM356-Chs2-mNG-KanMX6, pHis3-mRuby2-Tub1-Ura3* | This study |
| YSP1019 | *ESM356-Chs2-mNG-KanMX6, pHis3-mRuby2-Tub1-Ura3, ∆elm1::3HA-Trp* | This study |
| YSP1002 | *ESM356-Chs2-mNG-KanMX6, pHis3-mRuby2-Tub1-Ura3, ∆gin4::3HA-Trp* | This study |
| YSP003 | *MATα ura3-52 lys2-801amber ade2-101ochre trp1∆63 his3∆200 leu2∆1 (*YPH500) | ^2^ |
| YSP004 | *MATa leu2 ADE2 ura3-52::URA3-lexA-op-LacZ his3 trp* (SGY37) | ^2^ |
| YSP997 | *ESM356-Cdc3-mCherry-Ura, ∆gin4::3HA-Trp* | This study |
| YSP1581 | *ESM356-Cdc3-mCherry-Ura ∆gin4::3HA-Trp pRS305-(Vector alone) cut with KasⅠ and integrated into Leu locus* | This study |
| YSP1582 | *ESM356-Cdc3-mCherry-Ura ∆gin4::3HA-Trp pRS305-pTEF-Gin4(FL)-GFP****-****tCyc1 cut with KasⅠ and integrated into Leu locus* | This study |
| YSP1583 | *ESM356-Cdc3-mCherry-Ura ∆gin4::3HA-Trp pRS305-pTEF-Gin4(K48A)-GFP-tCyc1 cut with KasⅠ and integrated into Leu locus* | This study |
| YSP1236 | *ESM356-Inn1-mNG-KanMX6, pHis3-mRuby2-Tub1-Ura3, ∆gin4::3HA-Trp, pRS305-(Empty vector)-cut with KasⅠ and integrated into leu locus* | This study |
| YSP1237 | *ESM356-Inn1-mNG-KanMX6, pHis3-mRuby2-Tub1-Ura3, ∆gin4::3HA-Trp, pRS305-pGin4-Gin4(FL)-tGin4-cut with KasⅠ and integrated into leu locus* | This study |
| YSP1238 | *ESM356-Inn1-mNG-KanMX6, pHis3-mRuby2-Tub1-Ura3, ∆gin4::3HA-Trp, pRS305-pGin4-Gin4(Kinase dead)-tGin4-cut with KasⅠ and integrated into leu locus* | This study |
| YSP1312 | *ESM356-Hof1-mNG-Kanmx6, pHis3-mRuby2-Tub1-Ura3, ∆gin4::3HA-Trp, pRS305-(Empty vector)-cut with Kas1 and integrated into leu locus* | This study |
| YSP1313 | *ESM356-Hof1-mNG-Kanmx6, pHis3-mRuby2-Tub1-Ura3, ∆gin4::3HA-Trp, pRS305-pGin4-Gin4(FL)-tGin4-cut with Kas1 and integrated into leu locus* | This study |
| YSP1314 | *ESM356-Hof1-mNG-Kanmx6, pHis3-mRuby2-Tub1-Ura3, ∆gin4::3HA-Trp, pRS305-pGin4-Gin4(Kinase dead)-tGin4-cut with Kas1 and integrated into leu locus* | This study |
| YSP001 | *MATa ura3-52 lys2-801amber ade2-101ochre trp1∆63 his3∆200 leu2∆1 (YPH499)* | ^2^ |
| YSP1086 | *YPH499-∆hof1::TRP1 pRS316-HOF1 ∆gin4::HIS3MX6 (MFY1474)* | ^3^ |
| YSP1357 | *YPH499 ∆hof1::TRP1 pRS316-HOF1 ∆gin4::HIS3MX6, pRS305-Empty Vector-cut with Kas1 and integrated into Leu locus* | This study |
| YSP1358 | *YPH499 ∆hof1::TRP1 pRS316-HOF1 ∆gin4::HIS3MX6, pRS305-pGin4-Gin4(FL)-tGin4-cut with Kas1 and integrated into Leu locus* | This study |
| YSP1359 | *YPH499 ∆hof1::TRP1 pRS316-HOF1 ∆gin4::HIS3MX6, pRS305-pGin4-Gin4(K48A)-tGin4-cut with Kas1 and integrated into Leu locus* | This study |
| YSP034 | *ESM356-Gin4-GFP-Hph* | This study |
| YSP1345 | *ESM356-Gin4-GFP-Hph ∆elm1::3HA-Trp* | This study |
| YSP1419 | *ESM356-Gin4-GFP-Hph, ∆elm1::3HA-Trp, Hsl1-GBP-KanMX6* | This study |
| YSP1651 | *ESM356-∆elm1::3HA-Trp ∆gin4::6HA NatNT2* | This study |
| YSP1665 | *ESM356-∆elm1::3HA-Trp ∆gin4::6HA NatNT2 pRS305 cut with KasⅠ and integrated into Leu locus* | This study |
| YSP1666 | *ESM356-∆elm1::3HA-Trp ∆gin4::6HA NatNT2 pRS305-pTEF-Gin4(FL)-GFP-tCyc1 cut with KasⅠ and integrated into Leu locus* | This study |
| YSP1667 | *ESM356-∆elm1::3HA-Trp ∆gin4::6HA NatNT2 pRS305-pTEF-Gin4(K48A)-GFP-tCyc1 cut with KasⅠ and integrated into Leu locus* | This study |
| YSP1658 | *ESM356-∆elm1::3HA-Trp ∆gin4::6HA NatNT2 pRS305 cut with KasⅠ and integrated into Leu locus Hsl1-GBP-KanMX6* | This study |
| YSP1652 | *ESM356-∆elm1::3HA-Trp ∆gin4::6HA NatNT2 pRS305-pTEF-Gin4(FL)-GFP-tCyc1 cut with KasⅠ and integrated into Leu locus Hsl1-GBP-KanMX6* | This study |
| YSP1653 | *ESM356-∆elm1::3HA-Trp ∆gin4::6HA NatNT2 pRS305-pTEF-Gin4(K48A)-GFP-tCyc1 cut with KasⅠ and integrated into Leu locus Hsl1-GBP-KanMX6* | This study |
| YSP1737 | *ESM356-Gin4-GFP-Hph, Hsl1-∆ka1-GBP-KanMX6* | This study |
| YSP1738 | *ESM356-Gin4-GFP-Hph ∆elm1:3HA-Trp Hsl1-∆ka1-GBP-KanMX6* | This study |
| YSP1590 | *ESM356-Gin4-GFP-Hph pHis3-mRuby2-Tub1-Ura3* | This study |
| YSP1594 | *ESM356-Gin4-GFP-Hph ∆elm1::3HA-Trp, pHis3-mRuby2-Tub1-Ura3* | This study |
| YSP1411 | *ESM356-Gin4-GFP-Hph, ∆elm1::3HA-Trp Shs1-GBP-KanMX6* | This study |
| YSP1415 | *ESM356-Gin4-GFP-Hph, ∆elm1::3HA-Trp, Bud4-GBP-KanMX6* | This study |
| YSP1423 | *ESM356-Gin4-GFP-Hph, ∆elm1::3HA-Trp, Bni5-GBP-KanMX6* | This study |
| YSP119 | *ESM356-Hsl1-GFP-Hph* | This study |
| YSP1485 | *ESM356-Hsl1-GFP-Hph ∆elm1::3HA-Trp* | This study |
| YSP1605 | *ESM356-Hsl1-GFP-Hph ∆elm1::3HA-Trp Shs1-GBP-KanMX6* | This study |
| YSP1606 | *ESM356-Hsl1-GFP-Hph ∆elm1::3HA-Trp Bud4-GBP-KanMX6* | This study |
| YSP1740 | *ESM356-Hsl1-GFP-Hph, ∆elm1::3HA-Trp Kcc4-GBP-KanMX6* | This study |
| YSP1745 | *ESM356-Hsl1-GFP-Hph, ∆elm1::3HA-Trp Bni5-GBP-KanMX6* | This study |
| YSP1560 | *ESM356-Hsl1-GFP-Hph, ∆elm1::3HA-Trp Gin4-GBP-KanMX6* | This study |
| YSP036 | *ESM356-Kcc4-GFP-Hph* | This study |
| YSP1482 | *ESM356-Kcc4-GFP-Hph ∆elm1::3HA-Trp* | This study |
| YSP1734 | *ESM356-Kcc4-GFP-Hph, ∆elm1::3HA-Trp Hsl1-GBP-KanMX6* | This study |
| YSP1730 | *ESM356-Kcc4-GFP-Hph, ∆elm1::3HA-Trp Shs1-GBP-KanMX6* | This study |
| YSP1732 | *ESM356-Kcc4-GFP-Hph, ∆elm1::3HA-Trp Bud4-GBP-KanMX6* | This study |
| YSP1747 | *ESM356-Kcc4-GFP-Hph, ∆elm1::3HA-Trp Bni5-GBP-KanMX6* | This study |
| YSP1518 | *ESM356-Kcc4-GFP-Hph ∆elm1::3HA-Trp Gin4-GBP-KanMX6* | This study |
| YSP1906 | *ESM356-Kcc4-GFP-Hph pHis3-mRuby2-Tub1-Ura3* | This study |
| YSP1908 | *ESM356-Hsl1-GFP-Hph pHis3-mRuby2-Tub1-Ura3* | This study |
| YSP1910 | *ESM356-Kcc4-GFP-Hph ∆elm1::3HA-Trp pHis3-mRuby2-Tub1-Ura3* | This study |
| YSP1912 | *ESM356-Kcc4-GFP-Hph ∆gin4::3HA-Trp pHis3-mRuby2-Tub1-Ura3* | This study |
| YSP1914 | *ESM356-Hsl1-GFP-Hph ∆elm1::3HA-Trp pHis3-mRuby2-Tub1-Ura3* | This study |
| YSP1916 | *ESM356-Hsl1-GFP-Hph ∆gin4::3HA-Trp pHis3-mRuby2-Tub1-Ura3* | This study |
| YSP1588 | *ESM356-Elm1-GFP-Hph pHis3-mRuby2-Tub1-Ura3* | This study |
| YSP1592 | *ESM356-Elm1-GFP-Hph ∆gin4::3HA-Trp pHis3-mRuby2-Tub1-Ura3* | This study |
| YSP032 | *ESM356-Elm1-GFP-Hph* | This study |
| YSP1346 | *ESM356-Elm1-GFP-Hph ∆gin4::3HA-Trp* | This study |
| YSP1410 | *ESM356-Elm1-GFP-Hph, ∆gin4::3HA-Trp Shs1-GBP-KanMX6* | This study |
| YSP1414 | *ESM356-Elm1-GFP-Hph, ∆gin4::3HA-Trp, Bud4-GBP-KanMX6* | This study |
| YSP1418 | *ESM356-Elm1-GFP-Hph, ∆gin4::3HA-Trp, Hsl1-GBP-KanMX6* | This study |
| YSP1735 | *ESM356-Elm1-GFP-Hph, Hsl1-∆ka1-GBP-KanMX6* | This study |
| YSP1736 | *ESM356-Elm1-GFP-Hph ∆gin4::3HA-Trp Hsl1-∆ka1-GBP-KanMX6* | This study |
| YSP1591 | *ESM356-Elm1-GFP-Hph ∆gin4::3HA-Trp Cdc3-mCherry-Ura* | This study |
| YSP1453 | *ESM356-Elm1-GFP-Hph ∆gin4::3HA-Trp, Shs1-GBP-KanMX6, Cdc3-mCherry-Ura* | This study |
| YSP1715 | *ESM356-Elm1-GFP-Hph ∆gin4::3HA-trp ∆hsl1::natNT1 Cdc3-mCherry-Ura* | This study |
| YSP1717 | *ESM356-Elm1-GFP-Hph ∆gin4::3HA-trp Shs1-GBP-KanMX6 ∆hsl1::natNT1 Cdc3-mCherry-Ura* | This study |
| YSP1757 | *ESM356-Elm1-GFP-Hph ∆gin4::3HA-trp Inn1-3xmCherry-His3MX6* | This study |
| YSP1759 | *ESM356-Elm1-GFP-Hph, ∆gin4::3HA-Trp Shs1-GBP-KanMX6 Inn1-3xmCherry-His3MX6* | This study |
| YSP1755 | *ESM356-Elm1-GFP-Hph ∆gin4::3HA-trp ∆hsl1::natNT1 Inn1-3xmCherry-His3MX6* | This study |
| YSP1754 | *ESM356-Elm1-GFP-Hph ∆gin4::3HA-trp Shs1-GBP-KanMX6 ∆hsl1::natNT1 Inn1-3xmCherry-His3MX6* | This study |
| YSP1724 | *ESM356-Elm1-GFP-Hph ∆gin4::3HA-trp Myo1-3xmCherry-His3MX6* | This study |
| YSP1726 | *ESM356-Elm1-GFP-Hph, ∆gin4::3HA-Trp Shs1-GBP-KanMX6 Myo1-3xmCherry-His3MX6* | This study |
| YSP1722 | *ESM356-Elm1-GFP-Hph ∆gin4::3HA-trp ∆hsl1::natNT1 Myo1-3xmCherry-His3MX6* | This study |
| YSP1721 | *ESM356-Elm1-GFP-Hph ∆gin4::3HA-trp Shs1-GBP-KanMX6 ∆hsl1::natNT1 Myo1-3xmCherry-His3MX6* | This study |
| YSP1589 | *ESM356-Gin4-GFP-Hph Cdc3-mCherry-Ura* | This study |
| YSP1593 | *ESM356-Gin4-GFP-Hph ∆elm1::3HA-Trp Cdc3-mCherry-Ura* | This study |
| YSP1595 | *ESM356-Gin4-GFP-Hph ∆elm1::3HA-Trp Hsl1-GBP-KanMX6 Cdc3-mCherry-Ura* | This study |
| YSP1599 | *ESM356-Gin4-GFP-Hph Hsl1-GBP-KanMX6 Cdc3-mCherry-Ura* | This study |
| YSP1489 | *ESM356-Gin4-GFP-Hph Hsl1-GBP-KanMX6 Myo1-ymScarlet-I* | This study |
| YSP1491 | *ESM356-Gin4-GFP-Hph ∆elm1::3HA-Trp Hsl1-GBP-KanMX6 Myo1-ymScarlet-I* | This study |
| YSP1627 | *ESM356-Gin4-GFP-Hph Myo1-ymScarlet-I-Nat* | This study |
| YSP1629 | *ESM356-Gin4-GFP-Hph ∆elm1::3HA-Trp Myo1-ymScarlet-I-Nat* | This study |
| YSP951 | *ESM356-Cdc3-mCherry-Ura* | This study |
| YSP1033 | *ESM356-Cdc3-mCherry-Ura, ∆elm1::3HA-Trp* | This study |
| YSP2374 | *ESM356-Cdc3-mCherry-Ura ∆swe1::His3MX6* | This study |
| YSP2376 | *ESM356-Gin4-GFP-Hph ∆elm1::3HA-Trp Cdc3-mCherry-Ura ∆swe1::His3MX6* | This study |
| YSP2377 | *ESM356-Gin4-GFP-Hph ∆elm1::3HA-Trp Hsl1-GBP-KanMX6 Cdc3-mCherry-Ura ∆swe1::His3MX6* | This study |
| YSP2380 | *ESM356-Cdc3-mCherry-Ura, ∆elm1::3HA-Trp ∆swe1::His3MX6* | This study |
| YSP2378 | *ESM356-Gin4-GFP-Hph Hsl1-GBP-KanMX6 Cdc3-mCherry-Ura ∆swe1::His3MX6* | This study |
| YSP1348 | *ESM356-Gin4-∆ka1-GFP pHis3-mRuby2-Tub1-Ura3* | This study |
| YSP1207 | *ESM356-Hof1-mNG pHis3-mRuby2-Tub1-Ura3, Gin4-∆ka1::3HA-Trp* | This study |
| YSP949 | *ESM356-Myo1-mNG-KanMX6 pHis3-mRuby2-Tub1-Ura3* | This study |
| YSP1018 | *ESM356-Myo1-mNG-KanMX6 pHis3-mRuby2-Tub1-Ura3, ∆elm1::3HA-Trp* | This study |
| YSP1006 | *ESM356-Myo1-mNG-KanMX6 pHis3-mRuby2-Tub1-Ura3, ∆gin4::3HA-Trp* | This study |

**References:**

1.	Pereira, G., Tanaka, T.U., Nasmyth, K., and Schiebel, E. (2001-11-15). Modes of spindle pole body inheritance and segregation of the Bfa1p–Bub2p checkpoint protein complex. The EMBO Journal *20*. 10.1093/emboj/20.22.6359.

2.	Sikorski, R.S., and Hieter, P. (1989/05/01). A system of shuttle vectors and yeast host strains designed for efficient manipulation of DNA in Saccharomyces cerevisiae. Genetics *122*. 10.1093/genetics/122.1.19.

3.	Meitinger, F., Palani, S., Hub, B., and Pereira, G. (2013). Dual function of the NDR-kinase Dbf2 in the regulation of the F-BAR protein Hof1 during cytokinesis. Mol Biol Cell *24*, 1290-1304. 10.1091/mbc.E12-08-0608.
