# Supplementary material for: Nim1-related kinases regulate septin organization and cytokinesis by modulating Hof1 at the cell division site
Source: eLife. 2026 Sep 9;14:RP106366. doi: 10.7554/eLife.106366 (PMC13557712; doi:10.7554/eLife.106366)
Supplement: Supplementary file 3 [file elife-106366-supp2.docx]

**Oligonucleotides used in this study:**

| **Primer number** | **Primer name** | **Sequence** |
| --- | --- | --- |
| P86 | Elm1-S1 | GATATTATTTTTTGAACGCCAGGTTAACAATAATTACTTAGCATGAAATGCGTACGCTGCAGGTCGAC |
| P87 | Elm1-S2 | GATATTATTTTTTGAACGCCAGGTTAACAATAATTACTTAGCATGAAATGCGTACGCTGCAGGTCGACTCCGACAGATATCATCCTGTAGTTTCATCTAATCGATGAATTCGAGCTCG |
| P88 | Elm1-S3 | CAGTAGAAGATGTAAAAACGTATCTGAACTTTGCAGATAATGGTCAAATACGTACGCTGCAGGTCGAC |
| P110 | Gin4-S1 | TTGATCAAGAAAGATATTCGCAGCACAATACAATAATAACATTCAAAATGCGTACGCTGCAGGTCGAC |
| P111 | Gin4-S2 | GGCAGAACAAAACGAAGGAGACAAAACATGATTGCATTACATTAGCACTAATCGATGAATTCGAGCTCG |
| P112 | Gin4-S3 | TGGTCAATGAAGTTGAGAACGTCCTGAATAAGGAAGGCGTTCTACAAAAACGTACGCTGCAGGTCGAC |
| P113 | Hsl1-S1 | ACTCCTTTATTATTTGTTATTAATATTTTTTATTTTTACCACACGACATGCGTACGCTGCAGGTCGAC |
| P114 | Hsl1-S2 | AATTTTTCAAATTATTGTTGTATAATTATATAACATCTATATAGAATTTAATCGATGAATTCGAGCTCG |
| P115 | Hsl1-S3 | AAAAGTTTAATGATGATGTGGAGAGAGTAATTCGAAATGCCGGACGTTCACGTACGCTGCAGGTCGAC |
| P107 | Kcc4-S1 | ATAGGCTTGATAATAGTGCTGACGTAAATTACCAGAACTACTGCAGTATGCGTACGCTGCAGGTCGAC |
| P108 | Kcc4-S2 | GTTACATCGTATTGTCCATTTGGGGATCGATTATCCCTCCCTTTTTTTTAATCGATGAATTCGAGCTCG |
| P109 | Kcc4-S3 | TTGCTACAGAAATCCAAATTATTTTACAAAAAGAAGGTGTTTTGGACAAACGTACGCTGCAGGTCGAC |
| P90 | Hof1-S2 | TTTCTTTTATCAGAAAACTAGTAAAATTGATATACATCGAGATCAatcgatgaattcgagctcg |
| P91 | Hof1-S3 | GGATTAATTCCCTATAATTTCATTCAGCTACTGCATCAAGGTCTTcgtacgctgcaggtcgac |
| P698 | Inn1-S2 | TTCTATTTAAATATTAGTATTACAATAACAGCATGTTTCTCTTGTCATTAATCGATGAATTCGAGCTCG |
| P699 | Inn1-S3 | GGAAGAATTCGATGTCTCCTACAAGAAAAAGACCACCTCCAAGGCTCAGCCGTACGCTGCAGGTCGAC |
| P742 | Chs2-S2 | TGAGAAAAAAGAGGGAATGACGAGAAATTAGCTGAAAAATACTGGCATTAATCGATGAATTCGAGCTCG |
| P741 | Chs2-S3 | CATGGGAAGTCTCTAAATTAGACTTACCAAATGTTTTCCACAAAAAGGGCCGTACGCTGCAGGTCGAC |
| P855 | pRS305-Gin4-Fwd | GGCGGCCGCTCTAGAACTAGTCTGGTGTTAAAGCAGAAATCACG |
| P1021 | pRS305-Gin4-Rvs | atcgaattcctgcagcccgggCGGTTATACAAATAGTAATCAACACG |
| P856 | Gin4-K48A-Rvs | TGCTTTTGATATTACAGCAACTGCCGCCTCTTGT |
| P857 | Gin4-K48A-Fwd | CAAGAGGCGGCAGTTGCTGTAAATCAAAAGCAG |
| P60 | Shs1-S2 | TATTTATTTATTTATTTGCTCAGCTTTGGATTTTGTACAGATACAACTCAATCGATGAATTCGAGCTCG |
| P61 | Shs1-S3 | AAAAAAATGACACGTATACTGATTTAGCCTCTATTGCATCGGGTAGAGATCGTACGCTGCAGGTCGAC |
| P81 | Bud4-S2 | CTCTCAGTCATCAGATTATATGCTGTTTTCATTCCATTAATCACCTTCTAATCGATGAATTCGAGCTCG |
| P82 | Bud4-S3 | AGGAGGAAAAGACTCGAACAACCGGTCATAATTTGAAGCAAGATTTTAATCGTACGCTGCAGGTCGAC |
| P84 | Bni5-S2 | TCTGTCTTAATTTATAAATATTTATAACAACCCTTGGCGTAATGTAATCAATCGATGAATTCGAGCTCG |
| P85 | Bni5-S3 | GAAAGCAAATTGATCGTGGCGAAAATGGCCAATTTTGGATTGGAACTAAACGTACGCTGCAGGTCGAC |
| P906 | Hof1-(1-350 aa)-Fwd | ATCTGATCGAAGGTCGTGGGATCCCCATGAGCTACAGTTATGAAGCTTGTTT |
| P908 | Hof1-350-Rvs | TGCGGCCGCTCGAGTCGACCCGGTGTGGAATTTCTAAATTGTGTAGCCATG |
| P1251 | pET28a-bdSUMO-Gin4-FL-Rvs | CGAGTGCGGCCGCAAGCTTGCTATTTTTGTAGAACGCCTTCCTTATTCAG |
| P1254 | pET28a-bdSUMO-PIP2BD/KA1-Fwd | gccaTGCTGCACCAAACTGGAGGCGGCTCGCAGGCAAAGGAT |
| P340 | Gin4-Fwd-Y2H | ggttctggccccggtcagggaATGGCAATCAATGGTAACAGTA |
| P341 | Gin4-Rvs-Y2H | gatatcgaattcctgcagcccCTATTTTTGTAGAACGCCTTCC |
| P613 | Gin4-KID-290-Fwd | ggttctggccccggtcagggaGGATCCCAAAAATATCCAAGTATAAGAG |
| P1291 | Gin4-PIP2BD/KA1(1003)-Fwd | ggttctggccccggtcagggaGGATCCACCTGTGTCAAGGCTTGATC |
| P338 | Elm1-Fwd | ggttctggccccggtcagggaATGTCACCTCGACAGCTTATAC |
| P339 | Elm1-Rvs | gatatcgaattcctgcagcccCTATATTTGACCATTATCTGCA |
| P611 | Elm1-KID- 420Fwd | ggttctggccccggtcagggaGGATCCTCATCGTCCAGTGTGAACCC |
| P132 | Hof1-Y2H-Fwd | ggttctggccccggtcagggaATGAGCTACAGTTATGAAGCT |
| P133 | Hof1-Y2H-Rvs | gatatcgaattcctgcagcccTCAAAGACCTTGATGCAGTAG |
| P618 | Hof1-261-Rvs | gatatcgaattcctgcagcccTTCATCCGCTGCCGTGAATG |
| P619 | Hof1-350-Rvs | gatatcgaattcctgcagcccTGTGGAATTTCTAAATTGTGTAG |
| P620 | Hof1-334-Fwd | ggttctggccccggtcagggaGGATCCCCATTGCCATCTCCAGAAGT |
| P621 | Hof1-600-Rvs | gatatcgaattcctgcagcccGACTGGAAAACCTTCACTGGTG |
| P622 | Hof1-600-Fwd | ggttctggccccggtcagggaGGATCCGTCATTGAATATGCCAAGGC |
| P1179 | Gin4-KA1-S1 | GAGCCACCATTGAACAAATCTGTGCAAAAAATTAGGGAAAAAAATGCTGGCTAAcgtacgctgcaggtcgac |
| P1180 | Gin4-KA1-S3 | GAGCCACCATTGAACAAATCTGTGCAAAAAATTAGGGAAAAAAATGCTGGCcgtacgctgcaggtcgac |
| P1184 | Hsl1-KA1-S3 | AATAATACAGAAAATAACTACTCTAAAATACCAAAACCCTCAACGATTAAAcgtacgctgcaggtcgac |
| P63 | Myo1-S2 | TTTAAATAAAGGATATAAAGTCTTCCAAATTTTTAAAAAAAAGTTCGTTAATCGATGAATTCGAGCTCG |
| P64 | Myo1-S3 | TAGGCTCGAAAAATATTGATAGTAACAATGCACAGAGTAAAATTTTCAGTCGTACGCTGCAGGTCGAC |
| P1766 | Swe1-S1 | CGTAGTGCTGGGGAAAAGTAAACACACACAGGCGCACACGAGAACAGATGcgtacgctgcaggtcgac |
| P1767 | Swe1-S2 | GTGTGGGAAAAAAGTATGTAAATAAAACAAGGTTTTTTGTTCCATTTATCAatcgatgaattcgagctcg |
| P1764 | Swe1-Col1 | CAACAACACAGTTGAACATTG |
| P1765 | Swe1-Col2 | CCATCCTTCCCTTATTCTCAA |
| P21 | Elm1-Col1 | GATGCCGCTCAACAGAGGTTATG |
| P22 | Elm1-Col2 | CGCAATAGAAGACCACTAAACTC |
| P48 | Gin4-Col1 | GTTTTGCGCCAAGTTTCGTCT |
| P49 | Gin4-Col2 | TAAGGGTAAATATAATAGTAC |
| P50 | Hsl1-Col1 | CTCCCTTTGGATCGTAAGTTC |
| P51 | Hsl1-Col2 | ACCCCTCCAGGGTTGTATTTA |
| P46 | Kcc4-Col1 | ATGTTTGCCATGGTAGGTTGA |
| P47 | Kcc4-Col2 | TTTCTGCTATTACAATTAAAG |
| P334 | His3MX6-Int-Fwd | gcgcggctactagtcttact |
| P335 | His3MX6-Int-Rvs | tgtccaaagcgatggcaac |
| P1175 | Trp-Int-Fwd | TACCTAAGTAATCAGCACCAT |
| P1176 | Trp-Int-Rvs | GCCATTAATATGTTACCAAAT |
| P185 | Nat-Int-Fwd | ggctggaggtcaccaacgtca |
| P1523 | Nat-Int-Rvs | cctcccgcagggtgaagccgt |
