# Supplementary material for: Nim1-related kinases regulate septin organization and cytokinesis by modulating Hof1 at the cell division site
Source: eLife. 2026 Sep 9;14:RP106366. doi: 10.7554/eLife.106366 (PMC13557712; doi:10.7554/eLife.106366)
Supplement: Supplementary file 4 [file elife-106366-supp3.docx]

**Plasmids used in this study:**

| **Plasmid number** | **Description** | **Source** |
| --- | --- | --- |
| piSP23 | pFA6a-HIS3-MX6 | ^1^ |
| piSP14 | pYM25 (yeGFP-hphNT1) | ^2^ |
| piSP20 | pYM22 (3HA-klTRP1) | ^2^ |
| piSP185 | pYM17 (6HA-natNT2) | ^1^ |
| piSP659 | pDK88 (mNeongreen cassette. mNG-kanmx6) | Gift from Prof. Gislene Pereira |
| piSP1075 | pHA29-GBP-KAN | ^3^ |
| piSP31 | YIP211-cdc3-mCherry (URA3) | ^4^ |
| piSP34 | YIP204-cdc3-mCherry (TRP1) | ^5^ |
| piSP678 | pAFS125-GFP-TUB1 | ^6^ |
| piSP1224 | pHIS3p:mRuby2-Tub1+3"UTR::Ura3 | ^7^ |
| piSP1 | pRS305 | ^8^ |
| piSP1341 | pRS305-pGin4-Gin4(FL)-tGin4 | This Study^a^ |
| piSP1739 | pRS305-pGin4-Gin4(K48A)-tGin4 | This Study^a^ |
| piSP1614 | pRS305-pTEF-Gin4-GFP-tCYC1 | This Study^b^ |
| piSP1704 | pRS305-pTEF-Gin4 (K48A)-GFP-tCYC1 | This Study^b^ |
| piSP656 | pBPE39-1(3xmCherry-His3MX6 cassette) | Gift from Prof. Gislene Pereira |
| piSP112 | pMM5S-Y2H (Empty vector) | ^9^ |
| piSP113 | pMM6S-Y2H (Empty vector) | ^9^ |
| piSP635 | pMM5S-Gin4-FL (1-1142aa) | This Study |
| piSP645 | pMM6S-Gin4-FL (1-1142aa) | This Study |
| piSP632 | pMM5S-Elm1-FL (1-640aa) | This Study |
| piSP642 | pMM6S-Elm1-FL (1-640aa) | This Study |
| piSP685 | pMM5S-Gin4-KID (290-1142aa) | This Study |
| piSP691 | pMM6S-Gin4-KID (290-1142aa) | This Study |
| piSP683 | pMM5S-Elm1-KID (421-640aa) | This Study |
| piSP689 | pMM6S-Elm1-KID (421-640aa) | This Study |
| piSP568 | pMM5S-Hof1-FL (1-669aa) | This Study |
| piSP580 | pMM6S-Hof1-FL (1-669aa) | This Study |
| piSP1275 | pMM5S-Hof1(1-350aa) | This Study |
| piSP1277 | pMM6S-Hof1(1-350aa) | This Study |
| piSP1279 | pMM5S-Hof1(334-600aa) | This Study |
| piSP1281 | pMM6S-Hof1(334-600aa) | This Study |
| piSP1283 | pMM5S-Hof1(600-669aa) | This Study |
| piSP1285 | pMM6S-Hof1(600-669aa) | This Study |
| piSP1839 | pMM5S-Gin4-KA1(1003-1142aa) | This Study |
| piSP1844 | pMM6S-Gin4-KA1(1003-1142aa) | This Study |
| piSP65 | pET28a-6HIS-bdSUMO | ^10^ |
| piSP1094 | pGEX-5X1 | GE Healthcare^11^ |
| piSP1320 | pGEX-5X1-Hof1(1-350) | This Study |
| piSP1712 | pET28a-6HIS-bdSUMO-Gin4(KA1) | This Study |
| piSP1720 | pYM17-ymScarletI | This Study |

^a & b^ plasmids piSP1341, piSP1739, piSP1614 and piSP1704 were cut with KasI restriction enzyme and integrated into *Leu* locus.

**References:**

1.	Knop, M., Siegers, K., Pereira, G., Zachariae, W., Winsor, B., Nasmyth, K., and Schiebel, E. (1999). Epitope tagging of yeast genes using a PCR-based strategy: more tags and improved practical routines. Yeast *15*, 963-972. 10.1002/(sici)1097-0061(199907)15:10b<963::Aid-yea399>3.0.Co;2-w.

2.	Janke, C., Magiera, M.M., Rathfelder, N., Taxis, C., Reber, S., Maekawa, H., Moreno-Borchart, A., Doenges, G., Schwob, E., Schiebel, E., and Knop, M. (2004). A versatile toolbox for PCR-based tagging of yeast genes: new fluorescent proteins, more markers and promoter substitution cassettes. Yeast *21*, 947-962. 10.1002/yea.1142.

3.	Bertazzi, D.T., Kurtulmus, B., and Pereira, G. (2011). The cortical protein Lte1 promotes mitotic exit by inhibiting the spindle position checkpoint kinase Kin4. J Cell Biol *193*, 1033-1048. 10.1083/jcb.201101056.

4.	Fang, X., Luo, J., Nishihama, R., Wloka, C., Dravis, C., Travaglia, M., Iwase, M., Vallen, E.A., and Bi, E. (2010). Biphasic targeting and cleavage furrow ingression directed by the tail of a myosin II. J Cell Biol *191*, 1333-1350. 10.1083/jcb.201005134.

5.	Wloka, C., Vallen, E.A., The, L., Fang, X., Oh, Y., and Bi, E. (2013). Immobile myosin-II plays a scaffolding role during cytokinesis in budding yeast. J Cell Biol *200*, 271-286. 10.1083/jcb.201208030.

6.	Straight, A.F., Marshall, W.F., Sedat, J.W., and Murray, A.W. (1997-7-25). Mitosis in Living Budding Yeast: Anaphase A But No Metaphase Plate. Science *277*. 10.1126/science.277.5325.574.

7.	Markus, S.M., Omer, S., Baranowski, K., and Lee, W.L. (2015). Improved Plasmids for Fluorescent Protein Tagging of Microtubules in Saccharomyces cerevisiae. Traffic *16*, 773-786. 10.1111/tra.12276.

8.	Sikorski, R.S., and Hieter, P. (1989/05/01). A system of shuttle vectors and yeast host strains designed for efficient manipulation of DNA in Saccharomyces cerevisiae. Genetics *122*. 10.1093/genetics/122.1.19.

9.	Hatano, T., Lim, T.C., Billault-Chaumartin, I., Dhar, A., Gu, Y., Massam-Wu, T., Scott, W., Adishesha, S., Chapa, Y.L.B., Springall, L., et al. (2022). mNG-tagged fusion proteins and nanobodies to visualize tropomyosins in yeast and mammalian cells. J Cell Sci *135*. 10.1242/jcs.260288.

10.	Hatano, T., Palani, S., Papatziamou, D., Salzer, R., Souza, D.P., Tamarit, D., Makwana, M., Potter, A., Haig, A., Xu, W., et al. (2022). Asgard archaea shed light on the evolutionary origins of the eukaryotic ubiquitin-ESCRT machinery. Nat Commun *13*, 3398. 10.1038/s41467-022-30656-2.

11.	Mancini Lombardi, I., Palani, S., Meitinger, F., Darieva, Z., Hofmann, A., Sharrocks, A.D., and Pereira, G. (2013). Lre1 directly inhibits the NDR/Lats kinase Cbk1 at the cell division site in a phosphorylation-dependent manner. Curr Biol *23*, 1736-1745. 10.1016/j.cub.2013.07.032.
