# Supplementary material for: Nim1-related kinases regulate septin organization and cytokinesis by modulating Hof1 at the cell division site
Source: eLife. 2026 Sep 9;14:RP106366. doi: 10.7554/eLife.106366 (PMC13557712; doi:10.7554/eLife.106366)
Supplement: Supplementary file 5 [file elife-106366-fig2-data2.zip › Figure 2-Source data-2.pdf]

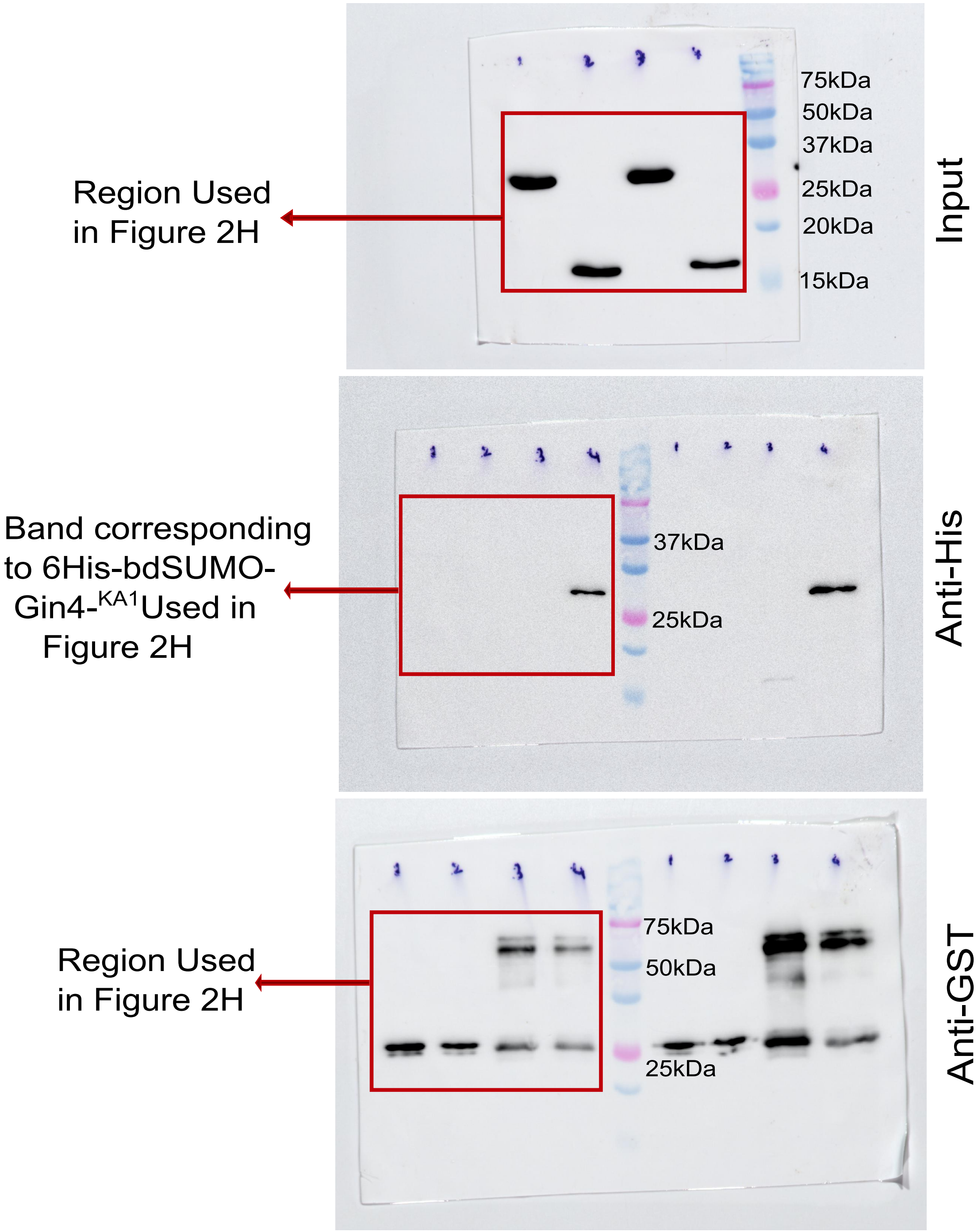

Figure 2-Source Data 2: Original uncropped western blot images corresponding to Figure 2H. Red boxes indicate the regions shown in the figure. The anti-His blot shows the band corresponding to 6×His-bdSUMO-Gin4-^{KA1}. Prestained molecular weight markers were used for protein size estimation and visualization.
