# Supplementary material for: Nim1-related kinases regulate septin organization and cytokinesis by modulating Hof1 at the cell division site
Source: eLife. 2026 Sep 9;14:RP106366. doi: 10.7554/eLife.106366 (PMC13557712; doi:10.7554/eLife.106366)
Supplement: Peer review history

# Editor report: eLife Assessment

**DOI:** 10.7554/eLife.106366.3.sa0

**Authors:** Alphee Michelot

**Affiliations:** Mechanobiology Institute Singapore

**Keywords:** Convincing, Valuable

In this **valuable** study, Bhojappa et al. investigate the function of four septin-associated kinases (Elm1p, Gin4p, Hsl1p and Kcc4p) in controlling septin ring stability, actomyosin ring organization and constriction, and cytokinesis in budding yeast. The evidence supporting the conclusions is **convincing**. The results are a meaningful addition to previously published work and provide a clearer picture of a complex mechanism involving proteins of partially redundant functions.

# Referee report: Reviewer #1 (Public review)

**DOI:** 10.7554/eLife.106366.3.sa1

Summary:

The authors wanted to better understand how the various septin-associated kinases contribute to septin organization and function in budding yeast. This question has been recently addressed by similar kinds of studies but there are still some open questions, particularly as regards to what extent the kinases may interact with and/or modify components of the contractile ring that drives cytokinesis.

Strengths:

This study uses sensitive imaging with good temporal and spatial resolution to monitor the localization of various proteins in living cells. Particularly informative is the use of a GFP/GFP-binding-protein "tethering" approach to ask if the requirement for one protein can be bypassed by physically tethering another protein to a third protein. Results from a yeast two-hybrid assay for measuring protein-protein interactions in vivo are buttressed by direct in vitro binding assays using purified proteins, which is important given the likelihood of "bridging" interactions between yeast proteins in the two-hybrid approach. The authors' conclusions are quite well supported by the data.

Weakness:

Ultimately, while the study provides some interesting and novel insights, we still don't understand which phosphorylation events on which proteins are important for the events occurring at the molecular level, so the advance in knowledge is somewhat incremental.

# Referee report: Reviewer #2 (Public review)

**DOI:** 10.7554/eLife.106366.3.sa2

Summary and strengths:

In this study, Bhojappa et al. investigate the roles of the septin-associated kinases Elm1, Gin4, Hsl1, and Kcc4 in septin organization and cytokinesis in budding yeast. Through quantitative analyses of kinase localization dynamics, septin organization, actomyosin ring (AMR) constriction, and cell morphology, the authors demonstrate that Elm1 and Gin4 play particularly important roles in maintaining proper septin architecture and cytokinetic progression. The work further identifies an interaction between the Gin4 KA1 domain and the Hof1 F-BAR domain and provides evidence that several cytokinesis-related functions of Gin4 are independent of its kinase activity. Artificial tethering approaches further supports that spatial organization at the bud neck is critical for the execution of septin-dependent cytokinetic processes. The authors combine live-cell imaging, quantitative analyses, biochemical interaction assays, and genetic perturbations to build a comprehensive framework for understanding how these kinases contribute to cytokinesis.

Comments on revised version:

The revised manuscript has been substantially improved. The authors have carefully addressed the concerns raised during review by providing additional experiments, analyses, quantifications, clarifications, and improved presentation of the data. The conclusions are now well supported by the experimental evidence. The study advances our understanding of the mechanisms linking septin organization to cytokinesis and will be of interest to researchers studying septins, cell division, and cytoskeletal regulation.

Overall Assessment:

This work provides valuable mechanistic insight into the coordination of septin organization and cytokinesis by septin-associated kinases. The experiments are carefully executed, the analyses are thorough, and the conclusions are supported by the data presented. The manuscript represents a useful contribution to the fields of cytokinesis and septin biology.

# Referee report: Reviewer #3 (Public review)

**DOI:** 10.7554/eLife.106366.3.sa3

Summary:

The study by Bhojappa et al. brings new and interesting elements about the stability of the septin ring and the crosstalk between septin and actomyosin ring assemblies. The study focuses on the four kinases associated with the septin ring, Elm1p, Gin4p, Hsl1p and Kcc4p. Elm1 and Gin4 show the strong knock-out phenotypes, whereas Hsl1p and Kcc4p show the weak knock-out phenotypes. The Elm1p/Kccp1p and Gin4p/Hsl1p pairs show similar timing at the bud neck. While these kinases share redundant functions, Gin4 appears to have a unique interaction with the BAR domain protein Hof1, revealing a novel direct interaction between the septin and actomyosin rings. Interestingly, the kinase activity of Gin4 is not required for its role in septin organisation and AMR constriction. The last part of the manuscript shows an original protein tethering protocol used to show that Hsl1 and its membrane binding ability are required for phenotype rescue of gin4null cells.

Comments on revised version:

I thank the authors for their thorough and thoughtful response to my review. The revised manuscript clearly reflects their efforts to provide rigorous and high-quality science. Addressing the concerns raised in the review required significant effort, but the improvements in the manuscript make it clear that the work was well worth it.

# Author comment: Author response

**DOI:** 10.7554/eLife.106366.3.sa4

**Authors:** Bindu Bhojappa, Anubhav Dhar, Bagyashree VT, Jayanti Kumari, Freya Cardozo, Vaseef Rizvi, Deepthi Guturu, Saravanan Palani

**Affiliations:** Indian Institute of Science Bangalore India; Indian Institute of Science Bangalore India; Indian Institute of Science Bangalore India; Indian Institute of Science Bangalore India; Indian Institute of Science Bangalore India; Indian Institute of Science Bangalore India; Indian Institute of Science Bangalore India; Indian Institute of Science Bangalore Bangalore India

The following is the authors’ response to the original reviews.

**Public Reviews:**

**Reviewer #1 (Public review):**

Summary:

The authors wanted to better understand how the various septin-associated kinases contribute to septin organization and function in budding yeast. This question has been recently addressed by similar kinds of studies but there are still some open questions, particularly as regards to what extent the kinases may interact with and/or modify components of the contractile ring that drives cytokinesis.

Strengths:

This study uses sensitive imaging with good temporal and spatial resolution to monitor the localization of various proteins in living cells. Particularly informative is the use of a GFP/GFP-binding-protein "tethering" approach to ask if the requirement for one protein can be bypassed by physically tethering another protein to a third protein. Results from a yeast two-hybrid assay for measuring protein-protein interactions in vivo are buttressed by direct in vitro binding assays using purified proteins, which is important given the likelihood of "bridging" interactions between yeast proteins in the two-hybrid approach. The authors' conclusions are quite well supported by the data.

Weaknesses:

A control for non-specific binding is missing from the in vitro binding assay. The figures suffer sometimes from the very small text in the labels, which obscures understanding. Ultimately, while the study provides some interesting and novel insights, we still don't understand which phosphorylation events on which proteins are important for the events occurring at the molecular level, so the advance in knowledge is somewhat incremental.

We thank the reviewer for highlighting the strengths of our imaging pipelines and protein-protein interaction data. We have now included appropriate controls for the in vitro binding assays, which demonstrate that the observed interactions are specific (Fig. 2H). We have also revised all figures to improve clarity, including increasing font sizes to enhance visibility across panels. We agree that mapping the specific phosphorylation sites regulated by these septin kinases would provide valuable mechanistic insights. However, only a few studies have addressed this direction so far (Mortensen et al., 2002; Asano et al., 2006 and Marquardt et al., 2024) [1-3]. The current study focuses on the interplay among septin-associated kinases and their role in regulation of the actomyosin machinery (AMR). In this context, we highlight several key findings:

(i) a molecular link between the septin kinase network and AMR through physical interaction between the KA1 domain of Gin4 and F-BAR domain of Hof1 (Fig. 2F-2H and S3H);

(ii) a kinase-independent role for Gin4 in coordinating septin organization and AMR dynamics (Fig. 3A-3E, S3F & S3G, S3I and 4F-4H);

(iii) a novel role for Hsl1 in regulating septins and the AMR downstream of Gin4 and Elm1, potentially through plasma-membrane binding (Fig. 4I-4K, 5A-5F, 8A-8G and S9H-S9J); and

(iv) crosstalk between Gin4 and Hsl1 that is independent of their role in the morphogenetic checkpoint (Fig. 6A-6D and S6A-S6C).

We have clarified this scope in the Discussion section and explicitly stated that mapping these phosphorylation sites will be an important direction for future work (Lines 623-626).

**Reviewer #2 (Public review):**

Summary:

In this paper, Bhojappa et al. provide insights into the function of septin-related kinases Elm1, Gin4, Hsl1, and Kcc4 in septin organization and actomyosin ring (AMR) structure and constriction. Their findings are both corroborative of and complementary to previous related studies.

First, the authors provide a comparative analysis of the dynamic localization of these kinases at the bud neck, as well as a comparative analysis of defects in septin localization, splitting dynamics, AMR constriction rates, and cell morphology in kinase deficient cells. They find that septin localization and splitting kinetics, as well as AMR constriction rates, are significantly perturbed in elm1∆ and gin4∆ mutants but remain largely unaffected in hsl1∆ and kcc4∆. A similar trend is observed in terms of cell morphology and viability.

Next, the authors focus on elm1∆ and gin4∆ cells, demonstrating that the residence time of the F-BAR protein Hof1 is significantly increased and defective in these mutants. Using yeast two-hybrid (Y2H) and in vitro binding assays, they show that the KA1 domain of Gin4 interacts with the F-BAR domain of Hof1, which may explain the cytokinesis-related functions of Elm1 and Gin4. Supporting this, they find that Gin4's role in septin localization, AMR constriction kinetics, and Hof1 bud neck localization is kinase-independent.

The authors then conduct a series of artificial tethering experiments given their bud neck localization is mostly interdependent. They first demonstrate that artificially tethering Gin4 to the bud neck rescues the morphology defects of elm1∆ cells, with the strongest rescue observed when Gin4 was forced to interact with Hsl1-an effect that was also kinase-independent. Additionally, artificial tethering of Hsl1 to the bud neck restores the morphology of elm1∆ cells in a KA1 domain-dependent manner, suggesting that Hsl1 functions downstream of Elm1 to maintain normal cell morphology. Consistently, artificial tethering of Elm1 to the bud neck in gin4∆ cells rescues morphology defects, as well as defects in Myo1 localization and AMR constriction, but only in the presence of full-length Hsl1. The rescue fails in the absence of Hsl1 or when using a version of Hsl1 lacking the KA1 domain, which supports the role of Hsl1 downstream to Elm1 in cytokinesis.

Strengths:

Altogether, this study offers valuable insights into the mode of cytokinesis regulation mediated by the septin-related kinases, mainly Elm1, Gin4, and Hsl1, and would be an important contribution to the field of septins and cytokinesis after addressing current weaknesses.

We thank the reviewer for the detailed summary and for highlighting the novel findings of our study.

Weaknesses:

(1) When assessing rescue of the elm1∆ phenotype, it needs to become clearer whether only morphology or also cytokinesis and septin organization are rescued.

To clarify the extent of rescue observed in *elm1Δ* cells, we extended our analysis beyond morphological parameters by quantifying septin organization and AMR constriction dynamics. These analyses now show that artificial tethering partially restores septin organization and AMR constriction kinetics in *elm1Δ* cells in addition to improving cell morphology. These results are now described in detail in Fig. 5 and lines 373-406.

(2) The quantification of the microscopy data does not always match up with the example images, and it's not always clear how the authors quantitatively analyzed their data.

We revised the manuscript to clearly outline the quantification methods used for microscopy data analysis and specified the statistical tests, number of cells analyzed, and number of experimental replicates in the figure legends. We also clarified the criteria used for phenotype scoring and quantification in the Materials and Methods section. In addition, We replaced representative images where necessary to accurately reflect the quantified data throughout the revised manuscript.

(3) The forced tethering data are key to the paper, but the lack of a summarizing table makes it difficult to grasp the full picture.

We agree with the reviewer and have now included a new summary table (Table 1) that compiles the results of all artificial tethering experiments presented in this study, including the percentage of rescue in cellular morphology observed upon forced tethering of these kinases to the bud neck, thereby providing a clearer overview of these experiments.

(4) Novel results and those confirming earlier results could be better distinguished.

We have improved the overall clarity of the manuscript to distinguish novel findings from the results that corroborate previous studies, and have cited the appropriate literature throughout the revised manuscript.

**Reviewer #3 (Public review):**

Summary:

The study by Bhojappa et al. brings new and interesting elements about the stability of the septin ring and the crosstalk between septin and actomyosin ring assemblies. The study focuses on the four kinases associated with the septin ring, Elm1p, Gin4p, Hsl1p, and Kcc4p. Elm1 and Gin4 show strong knock-out phenotypes, whereas Hsl1p and Kcc4p show weak knock-out phenotypes. The Elm1p/Kccp1p and Gin4p/Hsl1p pairs show similar timing at the bud neck. While these kinases share redundant functions, Gin4 appears to have a unique interaction with the BAR domain protein Hof1, revealing a novel direct interaction between the septin and actomyosin rings. Interestingly, the kinase activity of Gin4 is not required for its role in septin organisation and AMR constriction. The last part of the manuscript shows an original protein tethering protocol used to show that Hsl1 and its membrane binding ability are required for phenotype rescue of gin4null cells.

Strengths:

The combination of genetics, cell imaging, and biochemical characterization of proteinprotein interactions is attractive.

We thank the reviewer for recognizing the significance of our findings and for the helpful suggestions.

Weaknesses:

(1) Imaging and data analysis is the main weakness of this manuscript. The authors must avoid manual counting and selection when easy analysis software can be used to limit bias. Instead of presenting unclear statistics of "percentage phenotypes", they need to define clear metrics to offer meaningful phenotype analysis.

We agree that improving the quantitative rigour of the image analysis is essential for this study. Accordingly, we implemented a semi-automated image analysis workflow in the revised manuscript that defines reproducible metrics, such as aspect ratio, and reduces reliance on subjective phenotypic scoring. The inclusion of these parametric measurements enables clearer and more objective comparison of the rescued phenotypes.

(2) This manuscript examines a very complex mechanism with four kinases of overlapping function using new data and existing literature. A clearer picture/model at the end of the manuscript that synthesizes the current knowledge would be beneficial:

We incorporated a new representative model (Fig. 9) that integrates current knowledge in the field with our findings. This model highlights crosstalk among Elm1, Gin4, and Hsl1 as a key mechanism coordinating septin architectural transitions with AMR constriction during cytokinesis and is discussed in lines 520-536 of the revised manuscript.

We sincerely thank all the reviewers for their insightful comments. We incorporated new results in Fig. S3A, S3B, S4A-S4F, 5A-5F, 6A-6D, S6A-S6C, 8E, 8F, S10B and 9, along with Table 1 summarizing the artificial tethering experiments in the revised manuscript. We believe that these revisions have improved the rigor of our analyses and enhanced the overall clarity of the manuscript.

**Recommendations for the authors:**

**Reviewer #1 (Recommendations for the authors):**

(1) Line 70: " abnormal cytokinesis defects": this language is redundant, either "abnormal" or "defects" would suffice:

We thank the reviewer for identifying the redundant wording. We have rephrased the final paragraph of the Introduction section.

Please refer to line numbers 86-97.

(2) Currently, the final paragraph of the Introduction is an extensive, detailed summary of the results. This is unnecessary, as the Abstract and the Results sections summarize the results. Better would be a short statement of the questions addressed in the manuscript:

We thank the reviewer for this suggestion. We have revised the final paragraph of the Introduction to remove the detailed summary of results and instead outline the key questions addressed in this study. The revised paragraph now emphasizes the knowledge gap regarding how septin-associated kinases regulate septin organization and coordinate cytokinesis. Please refer to line numbers 86-97.

(3) In the Results, the wording of the heading associated with the first section is confusing. "and their defects during the cell cycle": it is not expected that the kinases themselves will have defects; defects may be observed in cells upon mutation of the kinases, but that is not clear from this wording:

We thank the reviewer for this comment. The heading has been revised from “and their defects during the cell cycle” to “and defects associated with their deletions” for improved clarity.

Please refer to line numbers 99-100.

(4) Lines 107-109 (" they may act as molecular signals for the transition and a trigger for crosstalk of cytokinesis") are redundant with earlier lines 104-105 (" suggesting them to be a possible trigger for septin remodelling"):

We have rephrased the text to improve clarity and avoid redundancy.

Please refer to line number 123-125.

(5) Lines 121-122: "Septin-associated kinases are believed to play an essential regulatory role": is the role essential, or is it regulatory? Since these kinases are not individually essential for cytokinesis, "essential" doesn't seem appropriate here:

This has been corrected in the revised manuscript.

(6) Figure 1 panels C and F: the font size is exceedingly small for the labels and should be greatly increased. This is true for multiple panels in Figures 2-5 and the Supplemental Figures as well:

We thank the reviewer for highlighting this issue. We have significantly increased the font sizes of the text and the x- and y-axis labels across all figures in the manuscript.

(7) The timing of mitotic spindle breakdown was used as a timepoint for comparison but it is not made clear in the manuscript how this was determined. Presumably, the mRuby2-Tub1 marker was visualized and the timepoint when the mitotic spindle separated into two discrete entities was called the "breakpoint" timepoint, but it would be important to better describe (and ideally show an example) of how that timepoint was determined. The only images I find with labelled tubulin are Tub1-GFP and these do not show spindle breakdown:

We thank the reviewer for raising this point. We used GFP-Tub1 (pAFS125-GFPTUB1) and mRuby2-Tub1 (pHIS3p:mRuby2-Tub1+3′UTR::URA3) plasmids to visualize spindle dynamics across the cell cycle, and in both cases defined the spindle breakpoint as time zero. As suggested, we have now included time-lapse images of Cdc3-mCherry and GFP-Tub1 in wild-type cells in Fig. S2D to illustrate the spindle breakpoint event used for temporal alignment. Corresponding changes have also been made in the Materials and Methods section to explicitly describe this analysis.

Please refer to line numbers 760-761.

(8) Line 165: "F-BAR protein Hof1, which senses and induces membrane curvature" and lines 170-172 "the F-BAR protein Hof1, which is known to be associated with septin hourglass and transit to AMR during split ring trigger". It is awkward to introduce the same protein twice, in two different ways, within a few lines of each:

We thank the reviewer for noting the redundant description. We have rephrased the text to introduce the F-BAR protein Hof1 in a more concise and streamlined manner while retaining the relevant functional information.

Please refer to line numbers 206-208.

(9) Throughout, it would be helpful to introduce more line breaks and organize the Results into smaller paragraphs:

We thank the reviewer for this suggestion. We have revised the layout of the Results section and introduced additional line breaks to improve readability.

(10) This is somewhat of a personal preference, but in the interest of transparency (and with the understanding that the 0.05 value is entirely arbitrary), would authors be willing to show actual P values in the figure panels rather than "**" or "ns", for example? Some readers may wish to apply a different standard of significance than 0.05, and not showing the P values makes this impossible. Furthermore, some readers (like this one) may interpret differently a P value of 0.044 versus "*" or 0.051 vs "ns":

We agree with the reviewer’s suggestion. To improve the transparency of the quantitative analyses, we have modified the graphs across all main and supplementary figures to display the “actual p-values” and specified the corresponding statistical tests in the figure legends, with significance indicated by asterisks. An example is provided in the attached image showing the residence time of Inn1-mNG in wild-type and *gin4*Δ cells complemented with kinase-active and kinase-dead Gin4 constructs (Fig. 3E in the revised manuscript). For this analysis, significance was assessed using the nonparametric Kruskal-Wallis statistical test.

(11) It is written that the authors "performed a Yeast Two Hybrid screen to find novel interacting partners at the bud neck" but I do not find evidence anywhere of a "screen" being performed, i.e., an unbiased search of many proteins to find a few interactors. Instead, it appears that the authors performed a two-hybrid assay to visualize interactions between a small, specific set of proteins. "Screen" should be replaced with "assay", as is currently the case in the Methods section. It is probably also valuable to point out in the text that using a yeast two-hybrid assay to assess interactions between yeast proteins has the caveat that any interactions observed could be indirect, as they may be "bridged" by endogenous yeast proteins.

We thank the reviewer for raising this point. Our Yeast Two-Hybrid experiments were performed using a small subset of septin-associated proteins rather than as an unbiased screen. Accordingly, we have replaced the term “screen” with “assay” throughout the revised manuscript and in the Materials and Methods section.

Please refer to line 241.

We also agree with the limitations inherent to this assay and have now explicitly stated it in the revised manuscript, please see lines 248-255.

(12) Panel 2H: I do not understand what the middle (as opposed to the top and bottom) blot segment represents. It is labelled "anti-HIS", like the one above it, but it is not associated with any molecular weight/ladder marker and I do not know what other species in the binding reaction in that lane would be recognized by the anti-HIS antibody. Perhaps the top segment is an "input" sample, and below it (in the middle segment) is what was bound to the beads. The figure legend is uninformative in this regard. Also, panel I in this figure is unnecessary to show, assuming that the bands shown in H are what I think they are. The blot makes the point without the need for quantification.

As suggested by the reviewer, we have added molecular weight markers for each blot panel showing the input and bead-bound fractions. The figure legend has also been updated accordingly to clearly describe the different blot segments and experimental conditions.

Please refer to figure legend 2H.

In addition, as suggested by the reviewer, we have removed the quantification graph corresponding to the in-vitro binding assay from the revised manuscript.

(13) The results in Figure 2H demonstrate that the Gin4-KA1 fragment is not non-specifically "sticky", because it does not bind GST alone, but there is no demonstration that binding by the Hof1 fragment is specific because there is no equivalent negative control for binding:

We thank the reviewer for this suggestion. We repeated the in-vitro binding assay using 6His-bdSUMO as a negative control alongside 6His-bdSUMO-Gin4^KA1^ to demonstrate binding specificity of the Hof1 fragment. The Hof1 N-terminal F-BAR fragment did not pull down the control 6His-bdSUMO fragment but specifically pulled down 6His-bdSUMO-Gin4^KA1^ under identical experimental conditions (Fig. 2H), confirming the specificity of the interaction between Hof1 F-BAR domain and Gin4KA1.

The corresponding text and results have been updated in the revised manuscript.

Please refer to Fig. 2H and lines 259-263.

(14) Line 257-258: "Localisation via Hsl1 is necessary to rescue the morphological defects exhibited by Δelm1 cells partially": what does "partially" refer to here? To the rescue, or the defects?

The term “partially” refers to the extent of rescue. Specifically, elongated cell morphology was rescued in 63.75% of the *elm1*Δ cell population, rather than in all cells, upon artificial tethering of Gin4-GFP to the bud neck via Hsl1-GBP.

(15) Lines 292-293: "can restore the morphological defects": this wording is unclear. "Restore" means "return to a former condition", which in this case would be normal cellular morphology, not defective cellular morphology. Similarly, see line 304: "While Myo1-3xmCherry mislocalisation was restored upon Elm1-GFP tethering": presumably the proper localization was restored, not the mislocalization:

In Lines 292-293, by phrase “can restore the morphological defects” was intended to indicate rescue of the elongated/clumped morphology associated with *gin4*Δ cells upon artificial tethering of Elm1-GFP to the bud neck via Shs1-GBP. We have now rephrased this sentence as: “can rescue the elongated/clumped phenotype exhibited by *gin4*Δ cells”.

Please refer to line numbers 481-482.

Similarly, in Line 304, the statement “While Myo1-3xmCherry mislocalisation was restored upon Elm1-GFP tethering” referred to rescue of the Myo1 mislocalization phenotype observed in *gin4*Δ cells. We have rephrased this sentence as: “However, Myo1-3xmCherry localization was restored to the bud neck upon artificial tethering of Elm1-GFP in gin4Δ cells”.

Please refer to lines 506-508 in the revised manuscript.

(16) Lines 295-296: "We find that Elm1-GFP tethering via Shs1-GBP, Bud4-GBP, and Hsl1-GBP" this should be "or", not "and":

Thank you for pointing this out. We have now corrected the text accordingly.

Please refer to line number 485.

(17) Lines 311 and 312 refer to "Inn1-3xmCherry lifetime" but previously Inn1 residence time was measured. Since fluorescence lifetime is a distinct kind of measurement/ assay, it seems important to clarify here what kind of experimental data are being referred to:

The term “Inn1-3xmCherry lifetime” was intended to describe the residence time of Inn1 at the cell division site, defined as the interval between the initial appearance of the Inn1 fluorescence signal and its complete disappearance during cytokinesis. For clarity and consistency, we have replaced the term “lifetime” with “residence time” in the revised manuscript.

Please refer to the line numbers 511 and 512.

(18) Discussion: "Septins are considered as the fourth cytoskeletal elements due to their extensive structural and functional diversity." This sentence is confusing, as it seems to imply that what defines a protein as being "cytoskeletal" is structural and functional diversity rather than anything to do with forming filaments, etc. The rest of this first paragraph of the Discussion also sounds like a summary of the background, is quite redundant with the Introduction, and should be shortened:

We thank the reviewer for this suggestion. We have rewritten the first paragraph of the Discussion to improve clarity, reduce redundancy with the Introduction, and better emphasize the main findings of the study.

Please refer to the line numbers 538-552 in the Discussion section.

(19) Lines 365-366: "Gin4 and Hof1 are synthetic lethal": this should be revised to "gin4∆ and hof1∆ are synthetic lethal". This is a good place to point out that standard yeast nomenclature inserts the ∆ symbol after the gene name, not before (as is done in *E. coli* genetics, for example):

We thank the reviewer for this suggestion. We have replaced “*Gin4* and *Hof1* are synthetic lethal” with “*gin4Δ* and *hof1Δ* are synthetic lethal” in the revised manuscript.

Please refer to line number 576.

In addition, we have now consistently placed the Δ symbol after the gene throughout the manuscript in accordance with standard yeast nomenclature.

(20) Line 399: "We also performed an extensive GFP-GBP screens": again, here "screen" implies that a large collection of genes/proteins were assayed, perhaps in an unbiased way, which does not accurately portray what was actually done, which was an extensive tethering study using GFP-GBP:

We thank the reviewer for this suggestion. We have replaced the term “GFP-GBP tethering screen” with “GFP-GBP tethering assay” throughout the revised manuscript. In addition, we have included a brief description of the specificity and functionality of GBP nanobody and its application in the GFP-GBP tethering strategy, extensively used in this study.

Please refer to line numbers 326-336.

**Reviewer #2 (Recommendations for the authors):**

Major points:

(1) Analysis of morphological defects of elm1∆ does not directly reflect the defects in cytokinesis and septin organization. For example, the deletion of SWE1 rescues the morphological defects of elm1∆ cells but not the cytokinesis or septin mislocalization (Bouquin et al., 2000). Considering this, the authors should address whether artificial tethering of Hsl1 to Gin4 in elm1∆ cells rescues the septin and cytokinesis defects or just the morphology. Is the role of Hsl1 in cytokinesis dependent on its role in the morphogenesis checkpoint? Can the authors comment on how much the defects observed by Hsl1 tethering to the bud neck may be a result of bypassing the morphogenesis checkpoint?:

We thank the reviewer for this important point. We performed time-lapse imaging of Cdc3-mCherry in strains where Gin4-GFP partially rescued the elongated phenotype of *elm1*Δ cells (63.75%) when tethered to the bud neck via Hsl1-GBP. Under these conditions, 64.29% of cells showed rescue of Cdc3-mCherry mislocalization, and Gin4 localization itself was restored to the bud neck in 57.85% of cells. We also examined Myo1-ymScarletI dynamics, while 76.92% of untethered *elm1*Δ cells displayed Myo1 mislocalization to the bud cortex, this was reduced to 11.36% upon Gin4-GFP tethering via Hsl1-GBP. Together, these results indicate that the morphological rescue observed in *elm1*Δ cells is accompanied by restoration of normal septin organization and AMR dynamics.

Previous work (Bouquin et al., 2000) [4] showed that Swe1 deletion rescues cell elongation in *elm1*Δ cells without restoring septin organization. Consistent with this, we found that 60.66% of *elm1*Δ *swe1*Δ cells exhibited a round morphology, but tethering of Gin4-GFP to the bud neck via Hsl1-GBP in *elm1*Δ *swe1*Δ background did not further enhance morphological rescue. These results suggest that the rescue of cellular morphology observed in our tethering experiments may, atleast in part, depend on Hsl1-mediated regulation of the morphogenesis checkpoint.

Importantly, despite the lack of additional morphological rescue, a clear restoration of septin localization was observed when Gin4-GFP was tethered to the bud neck via Hsl1-GBP in *elm1*Δ *swe1*Δ cells. Overall, these results suggest that while Hsl1-dependent morphogenesis checkpoint regulation may contribute to cell shape rescue, the restoration of septin organization is independent of Hsl1’s function in morphogenesis checkpoint and instead reflects a direct requirement for Gin4 and Hsl1 at the bud neck.

Please refer to Figures 5, 6, and S6 of the revised manuscript for these additional data.

(2) As suggested by the authors, the interaction of the Gin4-KA1 domain with the FBAR domain of Hof1 may explain the cytokinesis-related functions of Gin4. As an orthogonal approach, how does KA1 domain deletion of Gin4 affect cytokinesis and Hof1 bud neck localization?

We thank the reviewer for this suggestion. We first examined the bud neck localization of Gin4-*ka1*Δ-GFP in comparison with full-length Gin4-GFP. We observed that the localization kinetics of Gin4-*ka1*Δ-GFP were significantly altered relative to the full-length protein, with reduced recruitment and earlier removal from the bud neck. We also analyzed the localization kinetics of Hof1-mNG in both gin4-*ka1*Δ and *gin4*Δ cells. Our results show that Hof1-mNG displays increased residence time and altered accumulation kinetics during cytokinesis in both genetic backgrounds. Thus, loss of the KA1 domain phenocopies loss of the full-length Gin4 and is consistent with disruption of the physical interaction between Gin4 and Hof1.

Please refer to Figure S4 for these results in the revised manuscript.

(3) The authors state that "Elm1 and Kcc4 were present at lower abundance at the bud neck (Fig S1A-D) compared to the higher abundance of Gin4 and Hsl1, as observed in their fluorescence intensities (Figures S1B-C) ". This is not evident in the figures. The authors should show a quantification of how they judged abundance at the bud neck:

We thank the reviewer for this question. Quantification of septin kinase fluorescence intensity at the bud neck was performed using the established protocol for measuring protein accumulation kinetics described by Okada et. al. 2020 [5]. Time-lapse imaging for kinetic analysis of septin-associated kinases during bud emergence shown in Fig. S1A-S1D was carried out using a point-scanning confocal microscope with a 100×oilimmersion objective. Different laser intensities were required because the fluorescence signals of Kcc4 and Elm1 were comparatively weak and not readily detectable above cellular background under the imaging conditions used for Gin4 and Hsl1. The images shown in Fig. S1A-S1D are therefore displayed using differential contrast settings to facilitate visualization. We have now explicitly clarified this in the figure legend.

A more direct comparison of septin kinase abundance at the bud neck is now provided in Fig. S1E-F, where localization kinetics during the HDR transition/septin remodelling stage were captured using a laser-scanning spinning-disk microscope under similar imaging conditions. We have additionally included raw fluorescence intensity profiles during the HDR transition to better illustrate the relative abundance of these kinases at the bud neck during cytokinesis.

Please refer to Figures S1E and S1F in the revised manuscript for the updated images and quantitative analyses.

(4) How did the authors determine G1 and M-phase in the experiments shown in Figures S1A-D? Can the authors mark these phases on the timelapse images? Also, How do the authors explain the different behaviour of Cdc3 in graphs S1A-D among different strains?

We thank the reviewer for this comment. Cell cycle stages were initially inferred based on bud size, where kinase accumulation at the bud neck correspond to bud emergence (small bud, G1), and kinase disappearance coincided with septin splitting (large bud, M phase). However, we agree that accurate assignment of cell cycle stages would require specific cell cycle markers. To avoid confusion, we have removed the cellcycle-specific stage assignments from the Results section and describe the kinetics relative to t=0 (bud emergence).

The differential dynamics observed in the Cdc3-mCherry kinetic profiles likely reflect heterogeneity within the the cellular population. To address this, we combined the normalized fluorescence intensity profiles of Cdc3-mCherry from the strains expressing GFP-tagged septin kinases during bud emergence and have included this data as reference (Author response image 1).

**Author response image 1.** Plot showing spatiotemporal kinetics of Cdc3-mCherry in strains expressing either Elm1-GFP, or Gin4-GFP, or Hsl1-GFP, or Kcc4-GFP.

(5) Forced tethering based experiments are one of the key sets of experiments for this work, but it is difficult to have a comprehensive understanding of all the data considering how large the data set is and how dispersed it is in the supplemental and main figures (Figures 3-4-5 and Figures S4-S5-S6). It would be helpful to provide a table summarizing the tested forced tethering’s and the phenotypic outcome in the tested yeast strains (Wt/mutant):

We thank the reviewer for recognising the extensive dataset generated from the artificial tethering experiments and for suggesting the inclusion of a summary table. We have now added Table 1, which summarizes the proteins used in the GFP-GBP artificial tethering experiments, their genetic backgrounds, the total number of cells quantified across three independent replicates, and the phenotypic outcomes associated with bud neck tethering under each condition.

Please refer to Table 1 and lines 342, 361, 451, 453, 456 and 492 in the revised manuscript.

(6) In the introduction section, it would help the reader to provide more information on already known molecular roles of septin-associated kinases in septin organization and AMR. Later in the results section (i.e. Figure S1, S2, and S6), the authors extensively explain and show data that independently corroborate some earlier findings, which makes it difficult for the reader to distinguish novel findings from the repeated findings. I suggest shortening the text for the corroborative results, which will help to put more emphasis on their novel findings:

We thank the reviewer for this suggestion. We have now included the canonical roles of these four septin-associated kinases in the Introduction section of the revised manuscript.

Please refer to line numbers 79-85.

We have also revised sections describing corroborative findings and explicitly cited previous studies wherever relevant in the Results section to better distinguish previously established observations from the novel findings presented in this work.

Other minor comments:

(1) In Figure 1D-1F, also show the data for hls1∆ and kcc4∆ - which are shown in S3AC in the current version:

We have now included the Inn1-mNG residence time in *hsl1*Δ and *kcc4*Δ cells, alongside *elm1*Δ and *gin4*Δ in Fig. 1E.

Please refer to Fig. 1E in the revised manuscript.

(2) The authors should be more careful in interpreting their negative Y2H data in Figure 2F.

We have now explicitly discussed the caveats and inherent limitations of the Yeast Two-Hybrid assay in the manuscript.

Please refer to line numbers 248-255.

(3) Please provide quantification for Figure 5A, Figure S6I:

We have added quantitative analyses showing rescue of Cdc3-mCherry and Myo13xmCherry mislocalization in *gin4*Δ and *gin4*Δ *hsl1*Δ strains upon artificial tethering of Elm1-GFP to the bud neck via Shs1-GBP.

Please refer to Figures 8E, 8F, and S10B in the revised manuscript.

(4) In Figure S6: label is missing "∆" in front of hsl1:

We thank the reviewer for pointing out this error. We have corrected the labels accordingly.

(5) As common consensus on yeast gene nomenclature, I suggest the use of "gene∆" instead of "∆gene":

We thank the reviewer for this suggestion. We have now consistently placed the Δ symbol after deleted gene names throughout the manuscript in accordance with standard yeast nomenclature.

(6) Lines (535-536): min(distribution) and max(distribution) in the formula is confusing. Clarify it or if possible use "minimum value", "maximum value" instead:

We thank the reviewer for this suggestion. We have replaced the term “distribution” with “value” in the formula for protein accumulation kinetics analysis.

Please refer to the updated formula in the Materials and Methods section (Lines 752753).

(7) In line 86, "Dynamics of Septin-associated kinases and their defects during the cell cycle": Change the title as it is not clear what is meant by "their defects" given the discussed results under this title:

We thank the reviewer for this suggestion. We have revised the section heading from “and their defects during the cell cycle” to “and defects associated with their deletions”.

Please refer to line numbers 99-100.

(8) On the Hof1-mNG image (Fig2C), show the line used for the line scan profile. Additionally, a similar line-scan profile could be useful in Figure S3G:

As suggested by Reviewer 3, we removed the line-scan analysis from Figure 2 in the revised manuscript because Hof1 ring organization showed substantial heterogeneity across cells, making line-scan analysis difficult to interpret reliably.

**Reviewer #3 (Recommendations for the authors):**

Major points:

(1) The % phenotype units are terrible. With no explanation, we do not really know whether they represent the percentage of cells that have a particular phenotype, or whether they correspond to a metric that measures some deviation between normal and extreme phenotypes. I would strongly recommend using precise quantitative metrics systematically (i.e. intensities, aspect ratios, division times, etc.) to properly quantify phenotypes:

We thank the reviewer for suggesting the inclusion of precise quantitative metrics to assess phenotypic differences in the GFP-GBP tethering experiments. In the revised manuscript, we adopted quantification workflows that have been extensively validated and widely used in the literature, including those reported by Marquardt et al., 2024 (Fig. 7B and 7D) [2] from the Bi Lab. In response to the reviewer’s suggestion, we have now incorporated additional quantitative measurements, including cell area and aspect ratio (defined as the ratio of the cell’s major axis to the minor axis), for the experimental datasets presented in the manuscript.

In the main figures, we now include aspect ratio quantification, while additional parameters are provided for the reviewer’s reference. We also quantified the fluorescence intensity of tethered proteins at the large bud neck and present these data together with the aspect ratio analysis in Figure 4 for *elm1*Δ cells in which Gin4GFP is artificially tethered to the bud neck via Hsl1-GBP. These quantitative analysis corroborates our qualitative observations and further strengthens our conclusions. Please refer to Figures 4D and 4E as representative examples.

We have also changed the y-axis labels throughout the revised manuscript. For example, the y-axis in Fig. 7B is now labelled as “Cells exhibiting round morphology (%)”. Please refer to Fig. 4H, 4K, 7C, 8D, S5G, S6C, S7C, S9G and S9J for inclusion of aspect ratio quantification. Statistical analyses for the represented graphs were performed using Kruskal-Wallis nonparametric test, (N=3, n>150 cells/strain) (*: p<0.05, **: p<0.01, ****: p<0.0001, ns: p>0.05).

**Author response image 2.**

(2) Some quantitative analyses were performed manually where simple automated analysis should be performed to provide unbiased, accurate quantification:

We fully agree with the reviewer that automated image analysis approaches, such as segmentation-based methods, are generally preferred for minimizing bias in morphological quantification. However, *elm1*Δ and *gin4*Δ cells exhibit severe phenotypes, including pronounced elongation and clumping, which makes reliable automated segmentation technically challenging for accurate quantification of parameters such as aspect ratio and cell area. For this reason, we used manual annotation for these analyses, as this approach enabled accurate delineation of individual cell and reliable measurements of morphological parameters such as cell area and size across the datasets despite being more time-consuming.

Please refer lines 769-775 in the Materials and Methods section.

(3) The "tethering" data also lack clear quantification. The authors should properly quantify the average intensity of Hsl1-GFP at the bud neck in each condition and correlate the results with cell aspect ratios or any other relevant parameters. For example, when comparing elm1null and elm1null Bud4-GBP with elm1null Kcc4-GBP, the visual impression is that as much Hsl1-GFP protein is recruited to the bud neck, whereas the phenotypes are dramatically different:

We thank the reviewer for pointing this out. We have revised the image representation to facilitate clearer interpretation of the tethering experiments. In addition, we performed the key GFP-GBP tethering experiments using GBP-ymScarletI constructs, allowing direct visualisation of both the GFP-tagged protein and the GBP-tagged partner at the bud neck following tethering. We have included quantitative analyses of cellular morphology, raw fluorescence intensities of GFP-tagged proteins at the large bud neck, and the corresponding aspect ratio measurements for these updated datasets (Author response images 3, 4, 5). We have also included a summary table (Author response table 1) compiling these quantitative results for easier comparision.

(4) I am very confused by Figure 3 which shows normal localization of Gin4-GFP in elm1null cells and seems to contradict other claims in the manuscript. This is very problematic for the interpretation of most of the "tethering" data:

We understand the reviewer’s concern and have replaced the representative images of Gin4-GFP in *elm1*Δ cells in Figure 4B. Although Gin4-GFP is initially recruited to the presumptive bud neck during bud emergence in *elm1*Δ cells, it subsequently becomes mislocalized to the bud cortex during early cell cycle stages, resulting in reduced bud neck localization. As the cell cycle progresses, the Gin4-GFP signal at the bud neck decreases substantially in *elm1*Δ cells while remaining stable in wild-type cells until its departure prior to septin HDR remodelling (Fig. S5A-S5D).

(5) Figure 6 is neither explained in the text nor in its legend. Could the authors explain the model and offer a comprehensive picture of the current knowledge?

We have simplified the representative model to more clearly distinguish previously established knowledge from the findings presented in this study. Based on our results, We propose that Hsl1 functions both downstream of and in coordination with Elm1 and Gin4 to regulate septin stability and the timely execution of cytokinesis. Deletion of Elm1 disrupts the normal localization and crosstalk between Gin4 and Hsl1 at the bud neck, leading to septin mislocalization and misregulation of AMR dynamics, thereby revealing a previously uncharacterized role for Hsl1 in cytokinesis. The Results section has also been updated to reflect the revised model.

Please refer to Figure 9 and lines 520-536.

(6) Could the authors provide information about the double/triple mutant kinase phenotypes to clarify the overlap of functions among them?:

Barral et al., 1999 [6] reported that individual deletions of Hsl1 and Gin4 result in mild cytokinetic defects, whereas deletion of Kcc4 does not produce any striking phenotype compared to wild-type cells. In contrast, the *hsl1Δ gin4Δ kcc4Δ* triple mutant remains viable but exhibits severe morphological abnormalities, including branched chains of elongated cells with defective cell separation. These mutants also display aberrant septin organization at the bud neck, characterized by irregular patch-like structures. Analysis of double mutants (*hsl1Δ gin4Δ*, *gin4Δ kcc4Δ*, and *hsl1Δ kcc4Δ*) revealed intermediate phenotypes between the corresponding single and triple mutants, with the *hsl1Δ gin4Δ* combination showing the strongest defects. Together, these findings suggest that the Nim1-related kinases function redundantly to regulate Swe1 activity and maintain septin architecture at the bud neck.

Further supporting this model, Bouquin et al., 2000 [4] demonstrated that Elm1 operates independently of the Nim1-related kinases in controlling septin organization. The *hsl1Δ gin4Δ kcc4Δ elm1Δ* quadruple mutant exhibits severe septin localization defects and strong growth defects, in contrast to the *elm1Δ* single mutant, which primarily displays septin mislocalization from the bud neck to the bud cortex. These findings indicate that the combined activity of these kinases is essential for proper septin anchorage at the division plane and for assembly of the septin ring.

Importantly, the progressively stronger phenotypes observed in double, triple and quadruple mutants also suggest that these kinases retain partially specialized functions at the bud neck. Consistent with this framework, our results support a model in which Nim1-related kinases function redundantly to regulate septin architecture and cytokinesis, likely through modulation of the AMR machinery. Because the localization of these kinases appears interdependent, as reported previously (Marquardt et al., 2020; Marquardt et al., 2024) [2,7] and corroborated by our findings, interpretation of mutant phenotypes remains complex and future studies will be required to delineate their individual contributions more precisely.

Minor points:

(1) Abbreviations are not defined in the manuscript.

We have now expanded and defined all abbreviations throughout the manuscript.

(2) Some of the writing in the figures is too small. Please make sure that a minimal size of letters/numbers is respected:

We thank the reviewer for raising this issue. We have enlarged the figure labels and axis labels throughout the revised manuscript to improve readability.

(3) Figure 2D. I am not sure that the line scans bring any useful information as the rings in mutant cells are quite heterogenous. This panel is also not cited in the text. Please make sure that every panel is cited at least once:

We agree with the reviewer regarding the heterogeneity observed in the Hof1 ring organization in mutant cells and have therefore removed the line-scan analysis from the revised manuscript.

(4) Knocked-out genes are written incorrectly. Please use the usual yeast nomenclature:

We thank the reviewer for this suggestion. We have corrected the nomenclature for all deleted genes throughout the manuscript in accordance with standard yeast nomenclature.

**Author response image 3.** Artificial tethering of Gin4-GFP to the bud neck via Hsl1-GBP-ymScarletI rescues cellular morphology in *elm1*Δ cells. (A) Representative images showing artificial tethering of Gin4-GFP via Shs1-GBP-ymScarletI, Hsl1-GBP-ymScarletI, Bud4-GBPymScarletI and Bni5-GBP-ymScarletI in *elm1*Δ cells. DC*=Differential contrast. Scale bar5µm. (B) Bar graph representing the percentage of cells exhibiting round morphology in the indicated strains shown in (A), one-way ANOVA Tukey’s multiple-comparison test (**: p<0.01, ****: p<0.0001, ns: p>0.05), (N=3, wildtype: n=397, *elm1*Δ: n=408, *elm1*Δ-Shs1-GBPymScarletI: n=467, *elm1*Δ-Hsl1-GBP-ymScarletI: n=462, *elm1*Δ-Bud4-GBP-ymScarletI: n=401 and *elm1*Δ-Bni5-GBP-ymScarletI: n=317 cells). (C) Quantification of aspect ratios in the indicated strains shown in (A), Kruskal-Wallis nonparametric statistical test (***: p<0.001, ****: p<0.0001, ns: p>0.05), (N=3, n>170 cells/strain). (D) Graph depicting the raw fluorescence intensity of Gin4-GFP at the large bud in the indicated strains shown in (A), Kruskal-Wallis nonparametric statistical test (***: p<0.001, ****: p<0.0001, ns: p>0.05), (N=3, wildtype: n=166, *elm1*Δ: n=177, *elm1*Δ-Shs1-GBP-YmScarletI: n=176, *elm1*Δ-Hsl1-GBPymScarletI: n=188, *elm1*Δ-Bud4-GBP-ymScarletI: n=185 and *elm1*Δ-Bni5-GBP-ymScarletI: n=151 cells).

**Author response image 4.** Artificial tethering of Hsl1-GFP to the bud neck via septins or Nim1-related kinases rescues cellular morphology in *elm1*Δ cells. (A) Representative images showing the relocalization of Hsl1-GFP to the bud neck in *elm1*Δ cells via Shs1-GBP-ymScarletI and Gin4GBP-ymScarletI. Scale bar-5µm. (B) Bar graph representing the percentage of cells exhibiting round morphology in the indicated strains shown in (A), one-way ANOVA Tukey’s multiple comparison test (****: p<0.0001, ns: p>0.05), (N=3, wildtype: n=508, *elm1*Δ: n=418, *elm1*ΔShs1-GBP-ymScarletI: n=535 and *elm1*Δ-Gin4-GBP-ymScarletI: n=482 cells). (C) Quantification of the aspect ratios in the indicated strains shown in (A), Kruskal-Wallis nonparametric statistical test (*: p<0.05, ****: p<0.0001), (N=3, n>165 cells/strain). (D) Quantification of raw fluorescence intensity of Hsl1-GFP at the large bud in the indicated strains shown in (A), Kruskal-Wallis nonparametric statistical test (***: p<0.001, ****: p<0.0001), (N=3, wildtype: n=156, *elm1*Δ: n=166, *elm1*Δ-Shs1-GBP-ymScarletI: n=169 and *elm1*Δ-Gin4-GBP-ymScarletI: n=165 cells).

**Author response image 5.** Targeted localization of Kcc4-GFP to the bud neck via Hsl1-GBP-ymScarletI rescues cellular morphology in *elm1*Δ cells. (A) Representative images showing artificial tethering of Kcc4-GFP to the bud neck in *elm1*Δ cells via Shs1-GBP-ymScarletI, Hsl1-GBPymScarletI and Gin4-GBP-ymScarletI. Scale bar-5µm. (B) Quantitative analysis representing the percentage of cells exhibiting round morphology in the indicated strains shown in (A), oneway ANOVA Tukey’s multiple-comparison test (****: p<0.0001, ns: p>0.05), (N=3, wildtype: n=443, *elm1*Δ: n=489, *elm1*Δ-Shs1-GBP-ymScarletI: n=312, *elm1*Δ-Hsl1-GBP-ymScarletI: n=563 and *elm1*Δ-Gin4-GBP-ymScarletI: n=337 cells). (C) Quantification of the aspect ratios in the indicated strains shown in (A), Kruskal-Wallis nonparametric statistical test (****: p<0.0001, ns: p>0.05), (N=3, n>165 cells/strain). (D) Quantification for the raw fluorescence intensity of Kcc4-GFP at the large bud in the indicated strains shown in (A), Kruskal-Wallis nonparametric statistical test (**: p<0.01, ***: p<0.001, ****: p<0.0001), (N=3, wildtype: n=163, *elm1*Δ: n=168 *elm1*Δ-Shs1-GBP-ymScarletI: n=161, *elm1*Δ-Hsl1-GBP-ymScarletI: n=172 and *elm1*Δ-Gin4-GBP-ymScarletI: n=150 cells).

**Author response table 1.** Summary table showing rescue of elongated morphology in *elm1*Δ cells upon forced recruitment of Nim1-related kinases via septins or its related kinases tagged with GBPymScarletI.

Protein tagged with GFP	Protein tagged with GBP	Genetic background	Number of cells exhibiting round morphology	Number of cells exhibiting elongated/clumped morphology	Total number of cells	Percentage of cellular population exhibiting round morphology	Rescue of elongated or clumped morphology
Gln4	Shs1	elm1△	37	430	467	7.92	No
GIn4	Bud4	elm1△	52	349	401	12.96	No
Gln4	Hsl1	elm1 △	364	98	462	78.78	Yes
Gln4	BnI5	elm1△	0	317	317	0	No
Hsl1	Shs1	6/m1 △	333	202	535	62.24	Yes
Hsl1	Gln4	elm1 △	258	224	482	53.52	Yes
Kcc4	Shs1	elm1 △	24	288	312	7.69	No
Kcc4	Hsl1	eim1 △	402	161	563	71.40	Yes
Kco4	Gln4	eimf 1Delta	11	326	337	3.26	No

Additional changes:

The graph in Fig. S3D (revised preprint) has been updated to reflect a slight increase in the Chs2-mNG residence time in both the *elm1*Δ and *gin4*Δ strains, whereas our previous version indicated a delay only in the *gin4*Δ strain. Because the *elm1*Δ strain exhibited a more pronounced phenotype than the *gin4*Δ strain, we re-examined the analysis. The revised results show that the residence time of Chs2 during cytokinesis is modestly prolonged by approximately 2 minutes in both backgrounds. Accordingly, the graph and statistical analyses have been updated.

References:

(1) Mortensen, E.M., McDonald, H., Yates, J., and Kellogg, D.R. (2002). Cell Cycle-dependent Assembly of a Gin4-Septin Complex. Molecular Biology of the Cell 13, 2091-2105. 10.1091/mbc.01-10-0500.

(2) Marquardt, J., Chen, X., and Bi, E. (2024). Reciprocal regulation by Elm1 and Gin4 controls septin hourglass assembly and remodeling. J Cell Biol 223. 10.1083/jcb.202308143.

(3) Asano, S., Park, J.E., Yu, L.R., Zhou, M., Sakchaisri, K., Park, C.J., Kang, Y.H., Thorner, J., Veenstra, T.D., and Lee, K.S. (2006). Direct phosphorylation and activation of a Nim1-related kinase Gin4 by Elm1 in budding yeast. J Biol Chem 281, 2709027098. 10.1074/jbc.M601483200.

(4) Bouquin, N., Barral, Y., Courbeyrette, R., Blondel, M., Snyder, M., and Mann, C. (2000). Regulation of cytokinesis by the Elm1 protein kinase in *Saccharomyces cerevisiae*. Journal of Cell Science 113, 1435-1445. 10.1242/jcs.113.8.1435.

(5) Okada, H., MacTaggart, B., and Bi, E. (2021). Analysis of local protein accumulation kinetics by live-cell imaging in yeast systems. STAR Protoc 2, 100733. 10.1016/j.xpro.2021.100733.

(6) Barral, Y., Parra, M., Bidlingmaier, S., and Snyder, M. (1999 Jan 15). Nim1-related kinases coordinate cell cycle progression with the organization of the peripheral cytoskeleton in yeast. Genes & Development 13. 10.1101/gad.13.2.176.

(7) Marquardt, J., Yao, L.L., Okada, H., Svitkina, T., and Bi, E. (2020). The LKB1-like Kinase Elm1 Controls Septin Hourglass Assembly and Stability by Regulating Filament Pairing. Curr Biol 30, 2386-2394 e2384. 10.1016/j.cub.2020.04.035.
